# Fetal maturation revealed by amniotic fluid cell-free transcriptome in rhesus macaques

**DOI:** 10.1172/jci.insight.162101

**Published:** 2022-09-22

**Authors:** Augusto F. Schmidt, Daniel J. Schnell, Kenneth P. Eaton, Kashish Chetal, Paranthaman S. Kannan, Lisa A. Miller, Claire A. Chougnet, Daniel T. Swarr, Alan H. Jobe, Nathan Salomonis, Beena D. Kamath-Rayne

**Affiliations:** 1 Division of Neonatology, Cincinnati Children’s Hospital Medical Center, Cincinnati, Ohio, USA.; 2 Department of Pediatrics, University of Cincinnati School of Medicine, Cincinnati, Ohio, USA.; 3 Department of Pediatrics, University of Miami Miller School of Medicine, Miami, Florida, USA.; 4 Division of Biomedical Informatics, Cincinnati Children’s Hospital Medical Center, Cincinnati, Ohio, USA.; 5 California National Primate Research Center, UCD, Davis, California, USA.; 6 Division of Immunobiology, Cincinnati Children’s Hospital Medical Center, Cincinnati, Ohio, USA.; 7 Department of Bioinformatics, University of Cincinnati School of Medicine, Cincinnati Ohio, USA.; 8 Global Child Health and Life Support, American Academy of Pediatrics, Itasca, Illinois, USA.

**Keywords:** Reproductive Biology, Bioinformatics, Obstetrics/gynecology

## Abstract

Accurate estimate of fetal maturity could provide individualized guidance for delivery of complicated pregnancies. However, current methods are invasive, have low accuracy, and are limited to fetal lung maturation. To identify diagnostic gestational biomarkers, we performed transcriptomic profiling of lung and brain, as well as cell-free RNA from amniotic fluid of preterm and term rhesus macaque fetuses. These data identify potentially new and prior-associated gestational age differences in distinct lung and neuronal cell populations when compared with existing single-cell and bulk RNA-Seq data. Comparative analyses found hundreds of genes coincidently induced in lung and amniotic fluid, along with dozens in brain and amniotic fluid. These data enable creation of computational models that accurately predict lung compliance from amniotic fluid and lung transcriptome of preterm fetuses treated with antenatal corticosteroids. Importantly, antenatal steroids induced off-target gene expression changes in the brain, impinging upon synaptic transmission and neuronal and glial maturation, as this could have long-term consequences on brain development. Cell-free RNA in amniotic fluid may provide a substrate of global fetal maturation markers for personalized management of at-risk pregnancies.

## Graphical abstract

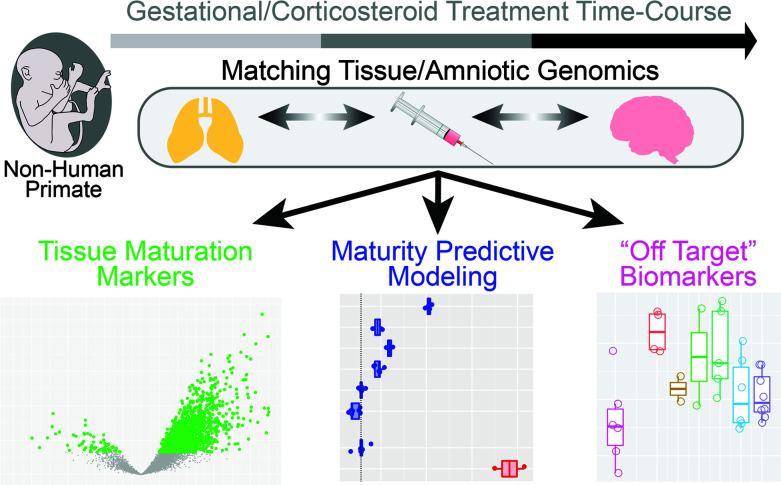

## Introduction

Preterm birth is responsible for 1 million infant deaths every year worldwide, with an even larger number of infants surviving with long-term respiratory and neurodevelopmental morbidities (1). Accurate assessment of fetal lung maturation could help guiding timely delivery of complicated pregnancies and administration of antenatal therapies that prevent morbidities associated with preterm birth, such as antenatal corticosteroids (2, 3). However, the traditional methods of assessment of fetal maturation are invasive, limited to the evaluation of fetal lung maturation, and do not provide information on the maturational state of other fetal organs (4, 5). The maturational state of the fetal brain is particularly important, given the continued growth and development of this organ through the third trimester and the increased risk of neurodevelopmental disabilities in preterm infants, even among those delivered in the late preterm period (34–36 weeks’ gestation) (6). Fetal maturity can also be accelerated clinically by administration of antenatal corticosteroids, which has been standard of care for pregnancies at risk of preterm delivery for more than 30 years (2). Antenatal corticosteroids have pleiotropic positive effects in the preterm fetus, particularly to the lung, but they also have potentially detrimental effects, particularly to brain development (7–9). In the fetal lung, corticosteroids promote thinning of the mesenchyme, facilitating gas exchange between the airspace and capillaries, and promote type II alveolar cell differentiation, leading to increased surfactant production; the end results of corticosteroids in the fetal lung are a more compliant lung with more effective gas exchange and decreased respiratory morbidity and mortality in preterm infants (10, 11). Fetal maturity testing that provides information on global maturity and the ability to longitudinally track fetal development and response to treatment would be an ideal tool for management of high-risk pregnancies and delivery planning.

To overcome the challenge of determining global fetal maturation, we evaluated the cell-free transcriptome of amniotic fluid for existing and potentially novel markers of fetal lung and brain maturation in a nonhuman primate model. Amniotic fluid contains fetal cell-free DNA, RNA, and proteins and reflects fetal status (12, 13). Furthermore, the composition of the amniotic fluid changes according to gestational age and fetal conditions (5, 14–17). Analyses of human pregnancies show that a portion of the fetal cell-free RNA in amniotic fluid can be traced to genes and biological processes associated with systems and organs that are not in direct contact with the amniotic fluid, such as the CNS (18). Moreover, the transcriptome of the amniotic fluid changes with advancing gestation, suggesting the presence of markers of fetal maturity (18). Cell-free RNA is of particular interest due to its presence not only in the amniotic fluid, but also, in smaller amounts, in the maternal serum. Circulating RNA correlates with changes in maternal-fetal development, as well as neurological diseases (19, 20). Analyses of the cell-free RNA in amniotic fluid could provide a foundation for targeted testing of fetal maturation markers in the maternal serum. While data from human samples raise intriguing questions regarding the presence of tissue-specific transcripts in amniotic fluid, these studies cannot directly evaluate transcriptome changes in fetal organs.

In this context, the amniotic fluid could provide information on the effects of antenatal corticosteroids beyond the lung and guide personalized treatment based on individual assessment of the maturational stage of various fetal organ systems. In a rhesus macaque model, we tested the hypothesis that changes to the amniotic fluid transcriptome reflect gene expression changes in the fetal lung and the fetal brain, and these changes correlate with intended (promotion of fetal maturity) and unintended effects from antenatal corticosteroid treatment.

## Results

### Fetal maturation is associated with broad coordinated transcriptional programs in lung, brain, and amniotic fluid.

**Figure 1:**
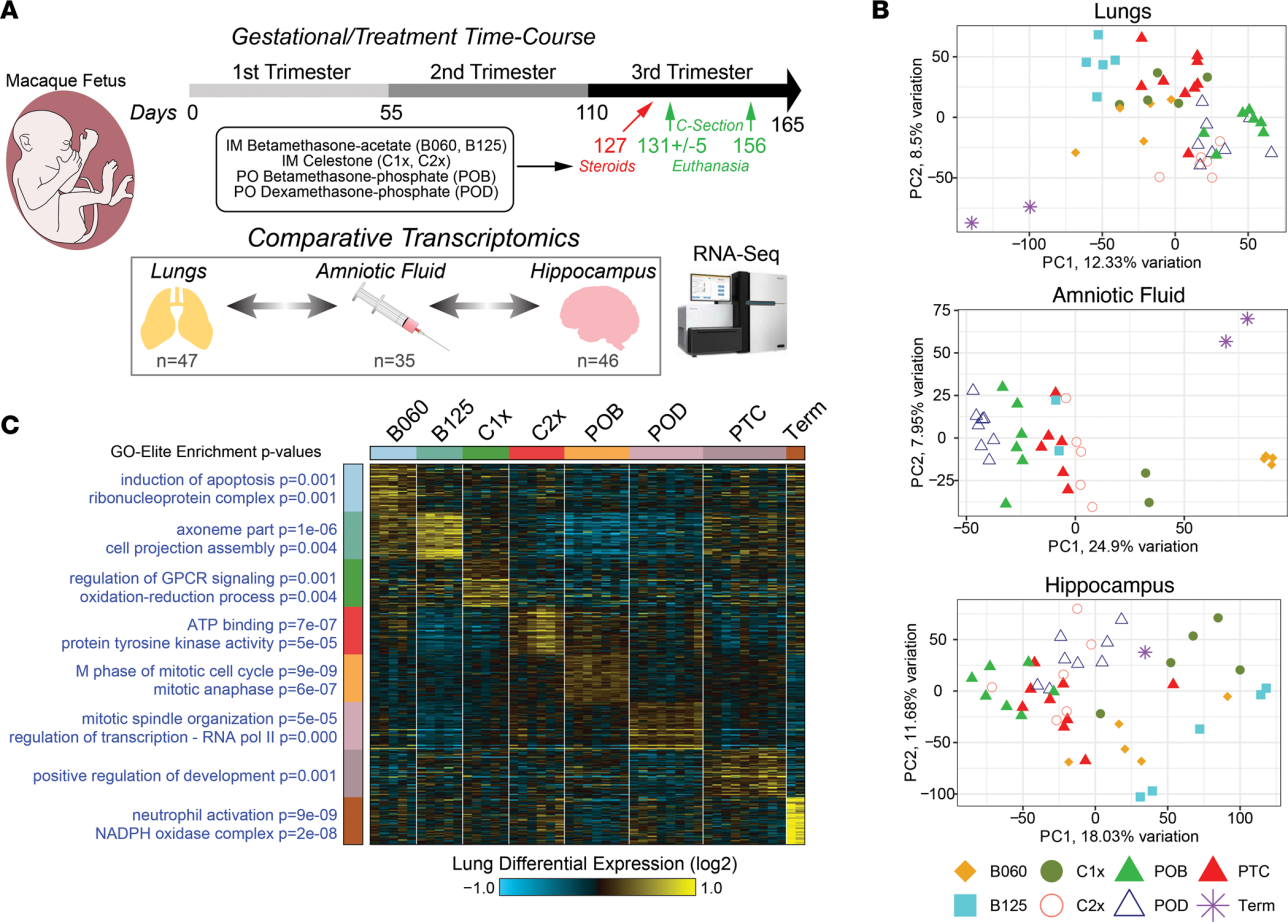
Evaluation of antenatal corticosteroids across tissues and amniotic fluid during rhesus gestation. (**A**) Study design for treatment and C-section of pregnant rhesus macaque females using indicated antenatal corticosteroid dosing. Amniotic fluid and brain (hippocampus) and lung tissue were collected from each fetus from either preterm (127–136 days) or term (156 and 157 days) gestation and analyzed by bulk RNA-Seq analysis. (**B**) PCA of all genes, following removal of strongly sex-associated genes and NOISeq batch-effect correction, for amniotic fluid, brain, and lungs (mean TPM ≥ 1). (**C**) Heatmap of the top most-specific marker genes for lung RNA-Seq for each treatment. Expression values were calculated as log_2_ fold changes relative to the mean of each row. The top GO-Elite Gene Ontology enrichment results are denoted to the left of each cluster, along with corresponding Fischer’s exact test enrichment *P* values. B060, i.m. betamethasone-acetate 0.06 mg/kg × 1 dose; B125, i.m. betamethasone-acetate 0.125 mg/kg × 1 dose; C1x, i.m. Celestone (betamethasone-acetate + betamethasone-phosphate) 0.25 mg/kg × 1 dose; C2x, i.m. Celestone 0.25 mg/kg × 2 doses; POB, oral betamethasone-phosphate 0.15 mg/kg × 3 doses; POD, oral dexamethasone-phosphate 0.15 mg/kg × 3 doses; and PTC, preterm control. *n* = 47 animals for lung; 35 animals for amniotic fluid and 46 animals for hippocampus.

We used a rhesus macaque model of fetal maturation to identify genes that are developmentally regulated from preterm to term gestation and the effect of antenatal corticosteroids in the amniotic fluid, fetal lung, and fetal brain transcriptome. Time-mated and ultrasound-dated pregnant rhesus macaque fetuses were delivered with intact membranes by cesarean section at preterm (~131 days) or near-term (~155 days) gestation, with full term being 165 days of gestation. The gestational age of delivery of preterm animals corresponds to about 32 weeks’ gestation in humans, when the lungs are at the saccular stage of development. Other groups of animals were treated with different formulations and doses of antenatal corticosteroids 5 days prior to delivery at preterm gestation (~131 days) (Figure 1A and Supplemental Table 1; supplemental material available online with this article; https://doi.org/10.1172/jci.insight.162101DS1). For each control time-point and antenatal corticosteroid treatment, multiple replicates were collected for matched fetal lung, fetal brain (hippocampus), and amniotic fluid; these were profiled using RNA-Seq, to allow for both differential and sample-level correlative analyses. As the specimens were from male and female fetuses and collected over multiple mating seasons, removal of strongly sex-associated genes and batch effects correction were applied to the resulting data, following gene-level transcript per million (TPM) quantification (Supplemental Table 2). When visualized using Principal Component Analysis (PCA), the transcriptomes of nearly all samples were readily separable by treatment groups and time points, with most intermixing in the hippocampus (Figure 1B). These data suggest a smaller transcriptional impact of antenatal corticosteroids on the fetal brain compared with the lungs, where corticosteroids are an established regulator of normal development. No clear sex bias was observed from this corrected data (Supplemental Figure 1A). Removing the term samples and considering additional informative principal components highlights clear treatment-specific transcriptomic impacts associated with term maturation (Supplemental Figure 1, B–D). Since these data suggest that each treatment regimen results in distinct molecular impacts, we further examined the presence of unique marker genes and enriched gene sets for each treatment group in lung, hippocampus, and amniotic fluid. Indeed, we found clear gene modules that defined each treatment group and term gestation in the fetal lung, with maturity at term most indicated by development of innate immunity (neutrophil activation, NADPH oxidase) (Figure 1C and Supplemental Table 3). Comparison of this maturity gene expression signature showed expected heterogeneity among the treatment groups, with less variability in term controls (Figure 1C, bottom cluster). Likewise, in the hippocampus, we found term maturation to be associated with myelination of neurons, with antenatal corticosteroid treatment differentially impacting Golgi-processing pathways (Supplemental Figure 2A and Supplemental Table 3). Overall, the data suggest that antenatal corticosteroid treatments have broad and unique impacts in the transcriptome of the amniotic fluid, lung, and hippocampus (Supplemental Figure 2B).

### Gestation gene expression differences are associated with cell type and tissue-specific cell-free RNAs.

**Figure 2:**
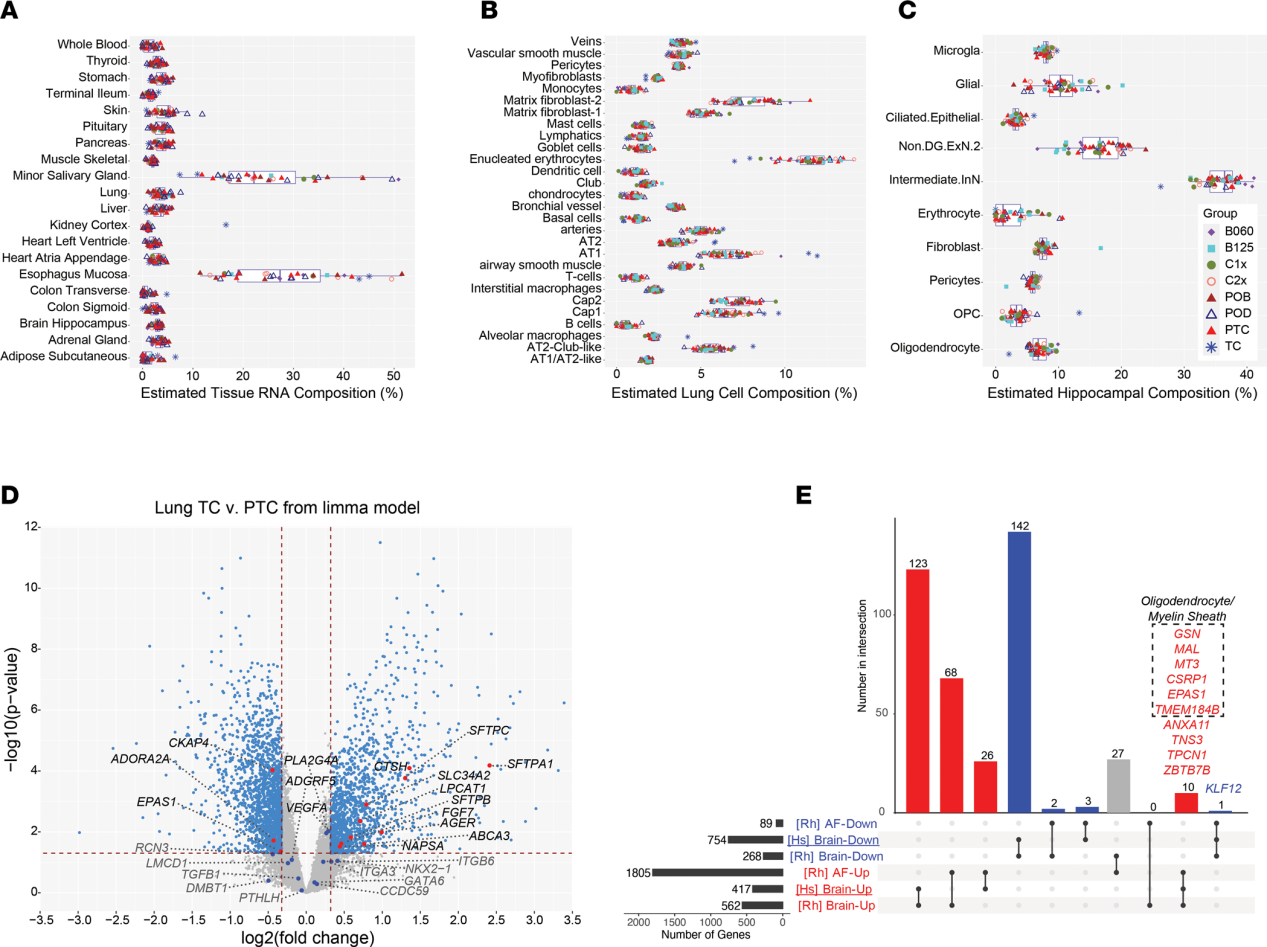
Amniotic fluid reflects tissue- and cell type–specific maturation programs. (**A**–**C**) Predicted relative frequency of Rhesus (**A**) amniotic fluid, (**B**) lung, and (**C**) hippocampus relative to appropriate reference tissue/single-cell collections, using deconvolution. (**A**) Predicted contribution of specific cell types to amniotic fluid relative to adult human tissue bulk RNA-Seq with GTEx, with only the most frequently detected tissues shown with specific estimates for all rhesus samples by treatment groups. (**B**) Predicted frequency of rhesus lung cell types, based on human neonatal, child, and adult scRNA-Seq samples. (**C**) Predicted frequency of Rhesus hippocampus cell-types using human fetal hippocampus cell population scRNA-Seq as a reference. (**D**) Volcano plot of differentially genes comparing the limma *P* value and fold change for all genes in term amniotic fluid versus preterm controls. Human term-induced genes from amniotic fluid defined previously are highlighted red (significant) or blue (nonsignificant) (fold ≥ 1.25 and limma *P* ≤ 0.05 adjusted for treatment year effects). Dashed reference lines mark fold change (vertical) and *P* value (horizontal) thresholds. (**E**) UpSet-style plot (marginal intersection counts) indicating overlap of differentially expressed genes in human neonatal versus midgestation hippocampus [Hs], compared with rhesus macaque term versus preterm hippocampus (25) and/or amniotic fluid [Rh]. Genes associated with oligodendrocyte cell identity and myelin sheath formation are highlighted. B060, i.m. betamethasone-acetate 0.06 mg/kg × 1 dose; B125, i.m. betamethasone-acetate 0.125 mg/kg × 1 dose; C1x, i.m. Celestone (betamethasone-acetate + betamethasone-phosphate) 0.25 mg/kg × 1 dose; C2x, i.m. Celestone 0.25 mg/kg × 2 doses; POB, oral betamethasone-phosphate 0.15 mg/kg × 3 doses; POD, oral dexamethasone-phosphate 0.15 mg/kg × 3 doses; PTC, preterm control; AT1/2, alveolar type 1/2; OPC, oligodendrocyte precursor cell; InN, inhibitory interneuron; and Non.DG.ExN.2, nondentate gyrus excitatory neuron 2. Data are presented as individual values; boxes represent median, 25th, and 75th percentiles; whiskers extend to ± 1.5 interquartile range. *n* = 47 animals (lung), 35 animals (amniotic fluid), and 46 animals (hippocampus).

While the rhesus macaque samples were profiled using bulk RNA-Seq, we investigated whether we could assess the contribution of specific cell types and associated mRNAs for each of the tissue/amniotic datasets. To this end, we identified markers for previously defined discrete cell populations from 2 large human single-cell RNA-Seq (scRNA-Seq) compendiums (lung and brain), as well as a large human pantissue bulk RNA-Seq library (GTEx), as a reference for cell-type frequency prediction (deconvolution) (Supplemental Table 4). Our in silico analysis of the potential tissue-of-origin for cell-free RNAs in amniotic fluid suggests that the most frequent cellular sources were the esophagus and salivary gland, with lower but detectable contributions from more than 15 other tissues, including lung and hippocampus, suggesting that fetal tissue cell-free RNA is abundant in the amniotic fluid (Figure 2A). Since placenta samples are not present in GTEx, it is possible that other organs that produce neurohormones may substitute for placenta hormone secreting cells in this analysis. We did not observe any statistical differences between term and preterm samples for estimated cell proportions in the amniotic fluid. Conversely, we applied an independent marker gene-based *Z* score analysis and found that lung and immune (whole blood, spleen, thyroid) markers were selectively increased in term relative to preterm, fitting with observations from prior studies (18, 21) (Supplemental Table 5 and Supplemental Figure 3A). Comparison of our fetal lung samples to a recent compendium of neonate, child, and lung scRNA-Seq (22) finds the largest contribution of mRNAs (deconvolution analysis) from matrix erythrocytes, matrix fibroblast, capillary endothelial (Cap1, Cap2), alveolar type 1 (AT1), and AT2-Club–like cells. We noted that, at term, there was a significant increase in the predicted frequency of AT1, AT2, and alveolar macrophage RNAs and a decrease in myofibroblast and erythrocytes, reflecting the alveolar epithelial differentiation that takes place in this period of lung development (Figure 2B). In addition to the noted regulation of myofibroblasts and macrophages, the marker gene *Z* score analysis predicted significant increases in monocytes, B cells, DCs, and mast cells at term and a decrease in airway smooth muscle cells and chondrocytes, supporting the findings from amniotic fluid (Supplemental Table 5 and Supplemental Figure 3B). Cell type predictions in the hippocampus, using a scRNA-Seq study of the developing human fetal hippocampus as a reference (23), found that intermediate inhibitory interneurons (InN), non-dentate gyrus excitatory neurons (Non.DG.ExN.2), and glial cells represent the principal sources of hippocampal RNA (Figure 2C). While we only have 1 term hippocampus sample in this cohort, term hippocampus RNAs showed a preliminary bias toward oligodendrocyte profiles, relative to all other gestational samples, as expected, given that the differentiation of oligodendrocyte progenitor cells into differentiated oligodendrocytes starts in the third trimester (Figure 2C and Supplemental Figure 3C) (24).

To determine whether transcripts identified as uniquely induced in term corresponded to previously identified markers of fetal maturation, we compared all differentially expressed genes in lung and amniotic fluid with our prior identified set of human term-induced amniotic fluid transcripts (18). In the rhesus macaque lung, 10 of 26 human amniotic fluid markers were overexpressed at term compared with preterm, several of which are well-defined markers of lung maturation and surfactant production (*SFTPA1*, *SFTPC*, *SFTPB*, *LPCAT1*, *FGF7*, *CTSH*) (Figure 2D and Supplemental Table 6). Although only 1 of these transcripts met the established significance threshold in macaque amniotic fluid, with the remainder just below the statistical threshold for significance, nearly all such markers matched the same direction of regulation, suggesting that their increased expression in the amniotic fluid reflects biological maturation of the lung (Supplemental Figure 4A). To assess term brain maturation impacts, we included additional age-matched term and preterm rhesus hippocampus bulk RNA-Seq samples from the PsychENCODE consortium (25). Differentially expressed genes in this independent cohort were highly concordant with those from the current study, with most common upregulated genes associated specifically with the myelin sheath Gene Ontology (GO) category (ToppFun *P =* 2 × 10^–7^, FDR corrected *- MAG*, *GJC2*, *CLDN11*, *PLP1*, *GFAP*, *MOBP*, *MBP*, *ERMN*) (Supplemental Figure 4B). To further verify that these samples are informative of primate hippocampus development, we compared these data with differentially expressed genes in human neonate/infant versus preterm (21 and 24 weeks) hippocampus, using bulk RNA-Seq from the Allen Brain Atlas (Supplemental Tables 7 and 8). We observed highly specific gene expression overlaps from human and rhesus macaque, with 123 upregulated and 142 downregulated genes conserved, suggesting that the identified targets reflect increased gene expression in the term hippocampus (Figure 2E). Importantly, amniotic fluid cell-free RNA was able to detect these same hippocampus human-rhesus shared myelin sheath term-induced transcripts (Figure 2E).

### Gestational maturation is associated with inflammatory signaling in the lung and amniotic fluid.

**Figure 3:**
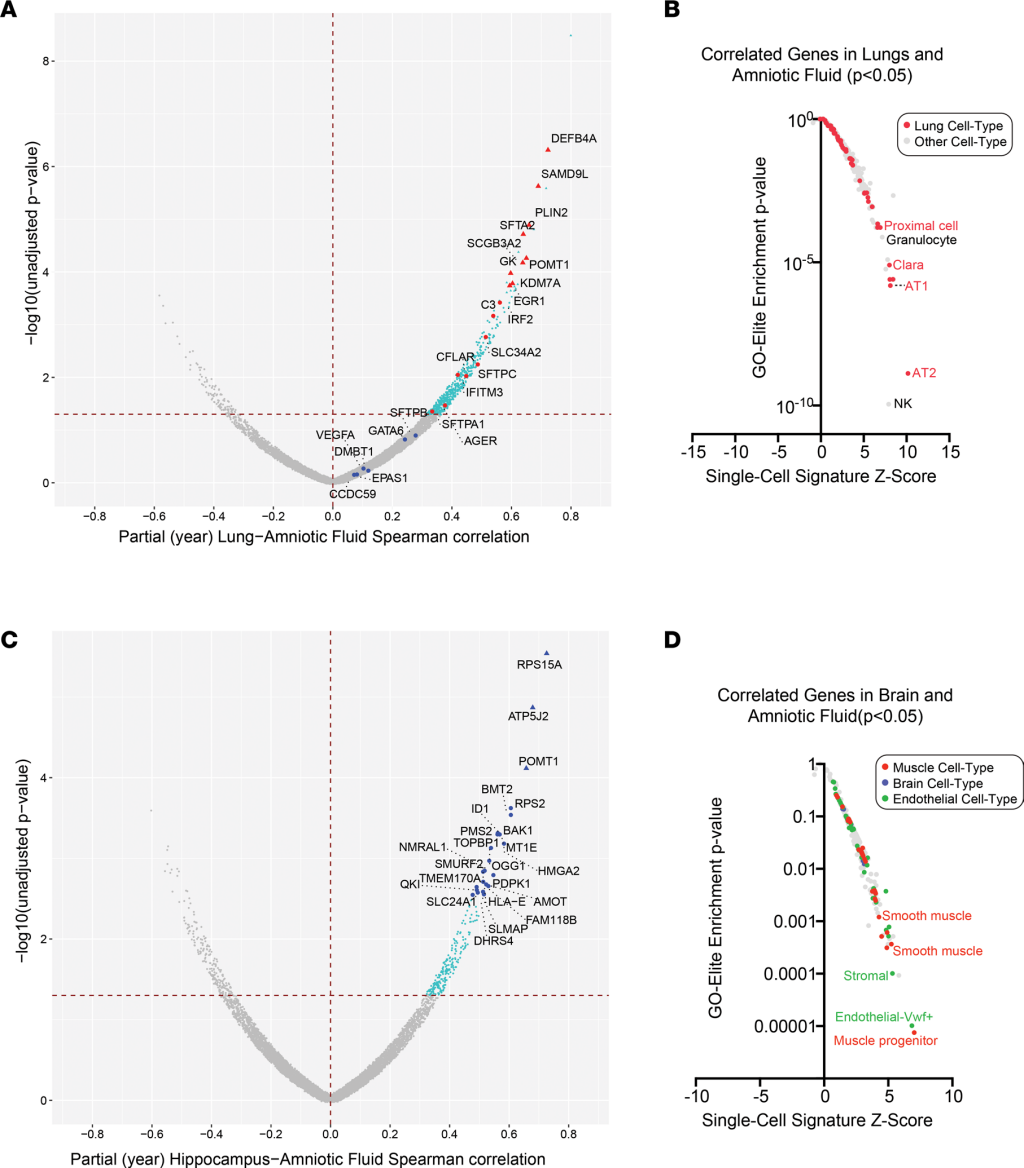
Detection of tissue maturation programs in amniotic fluid. (**A** and **C**) Volcano plot of gene expression correlation (Spearman’s rank, controlling for treatment year) from the same animals (treatment and controls) in (**A**) amniotic fluid and lungs or (**C**) amniotic fluid and hippocampus. Spearman *P* value and rank correlation are shown in the plot, with genes previously observed to be induced in human amniotic fluid, called out and designated by red (significant correlation) or blue (nonsignificant) in **A**. In **C**, the top significant genes (Spearman’s correlation ≥ 0.5 and *P* < 0.003) are designated in blue. Triangle markers denote FDR-adjusted *P* < 0.05. (**B** and **D**) Gene-set enrichment of all available single-cell, cell type signatures for (**B**) amniotic-fluid/lung positively correlated transcripts (*P ≤* 0.05) or (**D**) amniotic-fluid/brain positively correlated transcripts. Gene sets for lung associated with lung are specifically denoted in **B**, and those for muscle, brain, or endothelial (most frequently enriched) are denoted in **D**. *n* = 35 paired samples for lung with amniotic fluid and hippocampus with amniotic fluid.

A unique advantage of the study design of this sample cohort is matching paired tissue and amniotic fluid from the same animals. To identify genes with correlated expression across gestation and with antenatal steroid treatment, we identified genes with similar induction or repression profiles across tissue pairs, using Spearman’s rank, controlling for mating cohort. Transcripts positively correlated (*P ≤* 0.05) in amniotic fluid and lungs throughout gestation were dominated by established lung maturation marker genes, including *SFTPC*, *SFTPA1*, *SFTA2*, *SCGB3A2*, and others (Figure 3A and Supplemental Table 9). These lung/amniotic fluid–correlated transcripts were highly enriched in lung epithelial progenitor (Clara cells) and alveolar type 1 and type 2 cells markers, essential for gas exchange and surfactant production (Figure 3B), as well as in mRNAs associated with lung inflammation — in particular, monocyte activation and inflammatory transcriptional regulation *STAT3*, *PPARG*, *NFKB*, and *RELA* (Supplemental Figure 5, A and B, and Supplemental Table 10). These data support the model that advancement from preterm to near-term gestation is associated with activation of proinflammatory signaling and immune cell activation in the fetal lung and amniotic fluid, which is likely due to activation of inflammatory pathways in the maternal and fetal membranes with the approaching of parturition (26).

Unlike the lung, correlated transcripts in the hippocampus and amniotic fluid were associated with mixed effects — in particular, downregulated transcripts associated with different treatment and maturation impacts (Figure 3C and Supplemental Table 10). These correlated transcripts were highly enriched in vasculature markers, including muscle and endothelial cells (Figure 3D). Since these results do not reflect the shared oligodendrocyte progenitor markers we observed in brain and amniotic fluid, we surmised that these correlated transcripts were likely associated with broad brain vascular changes associated with growth.

### Amniotic fluid mRNAs can accurately predict fetal organ maturity.

**Figure 4:**
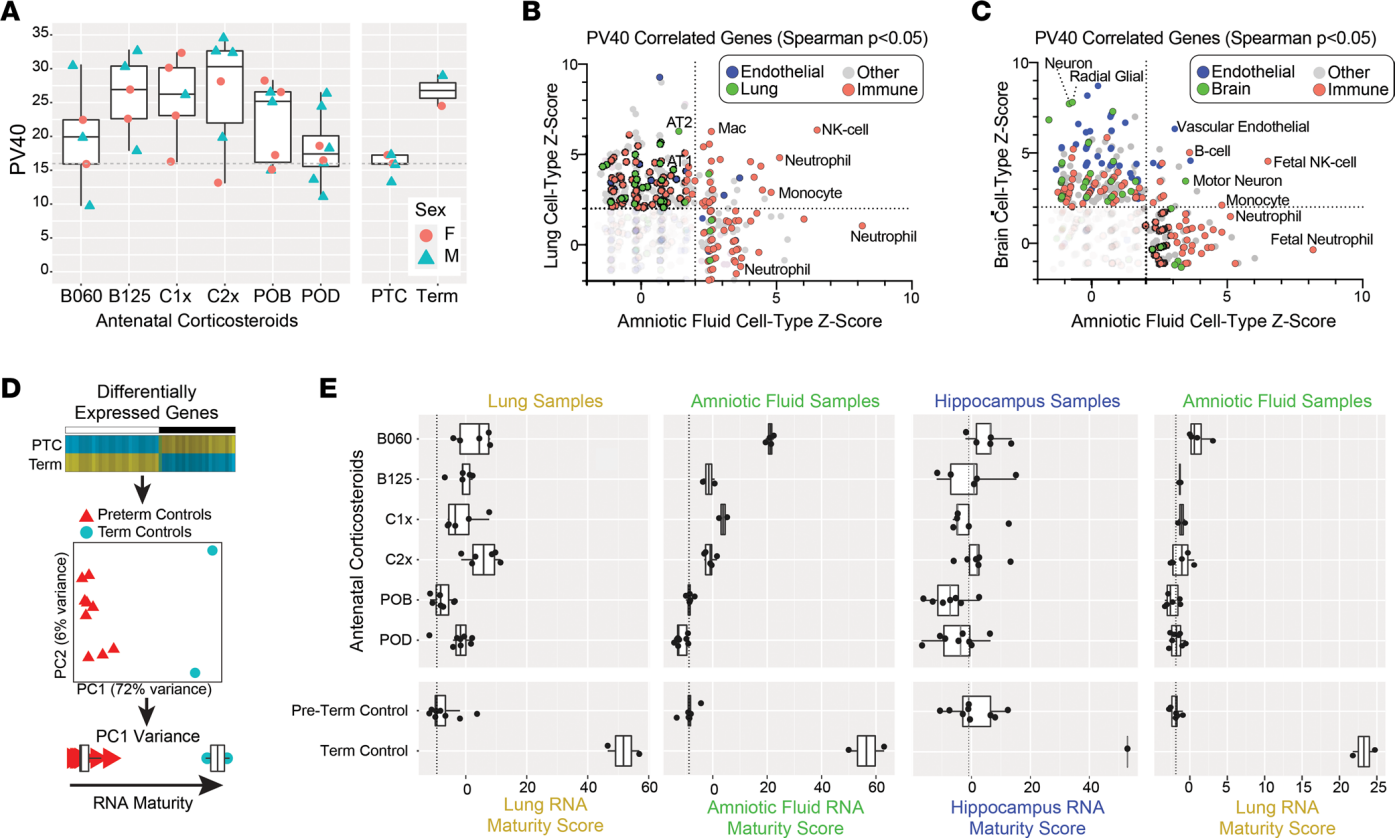
In silico tissue maturation analysis predicts optimal corticosteroid regimens from amniotic fluid. (**A**) Measurement of lung gas volume at 40 cmH_2_O on the pressure volume curve (PV40) was performed on fetal lungs immediately following euthanasia. Preterm samples had the lowest PV40 and term samples the highest, with substantial variation among antenatal corticosteroid treated animals. Male and female fetuses are separately indicated. (**B**) Statistical enrichment of gene sets from diverse single-cell data sets (*n* > 2,000) for lung and amniotic fluid transcripts that are positively correlated with PV40, across the collection of rhesus samples (2 outlier animals removed). Cell type signatures associated with endothelial cells, lung cells, immune, and cell type signatures associated with endothelial cells, lung cells, immune, and other annotated cell types are highlighted, along with significant enrichment results (*Z* score > 2, Fisher’s exact *P <* 0.05 [FDR corrected]) in either or both lungs and amniotic fluid. (**C**) Data plotted as in **B**, comparing statistically enriched single-cell signatures from brain and amniotic fluid samples. (**D**) RNA maturity scoring algorithm design. Differentially expressed genes for term versus preterm control animals are calculated (see Methods) and selected for PCA of term and preterm samples to capture the loadings for PC1 to compute scores for all samples using the PCATools predict function. (**E**) In silico maturity scores from each fetus lung, brain, or amniotic fluid sample are displayed according to treatment group using either the scoring schema from that same tissue (left 3 plots) or for amniotic fluid scored based on the lung scoring schema for lung PC1 loading genes. Data on **A** and **E** are presented as individual values, with box representing the median and the 25th and 75th percentile and whiskers extending to ± 1.5 IQR. *n* = 47 animals for lung, 35 animals for amniotic fluid, and 46 animals for hippocampus.

To evaluate maturity of fetal macaque lungs, with and without antenatal corticosteroid treatment, we measured lung gas volume at 40 cmH_2_O on the pressure volume curve (PV40) (Figure 4A). These data show that 25 of the 36 treated animals had PV40 measurements greater than the range of preterm controls (PTCs). Specifically, animals in the B125, C1x, C2x, and term control groups had mean PV40 measurement increases of greater than 9.5 units compared with animals in the PTC group (1-way ANOVA followed by Dunnett’s tests; *P =* 0.033 for C2x; Supplemental Table 11). Inclusion of sex as a covariate did not impact the results. Correlation of gene expression and PV40 found 727 genes in lungs, 234 genes in amniotic fluid, and 260 genes in hippocampus that correlate with lung functional maturation (unadjusted *P ≤* 0.05; Supplemental Table 12). However, only a handful of organ/PV40-correlated genes overlapped between lung with amniotic fluid (*ATF1*, *CBLB*, *CEACAM1*, *GPD1L*, *KRAS*, *LARP4B*, *MAL2*, *PLIN2*, *SEC24A*, *SERTAD3*) or hippocampus and amniotic fluid (*ADM*, *APPL2*, *ATF1*, *LYPLA1*, *NDUFA2*, *TMEM60*). Given that such gene programs likely reflect differing abundance of tissue-resident and nonresident cell populations, we examined each of these broader gene sets using gene set enrichment against marker genes for diverse scRNA-Seq–defined cell populations. Comparison of PV40-correlated transcripts in lung and amniotic fluid finds the common enrichment of mRNAs associated with monocyte, neutrophil, macrophage, and NK cells (Figure 4B and Supplemental Table 13). When considering only lung enriched cell type signatures, we observed a broad and specific enrichment of lung cell type signatures — in particular, of AT1 and AT2 cells, which undergo differentiation in late gestation in preparation for the initiation of breathing upon birth. Similarly, comparison of PV40-correlated transcripts in hippocampus and amniotic fluid found the common enrichment of monocyte and NK cell programs, in addition to B cell, vascular endothelial, and motor neuron programs (Figure 4C). Brain-specific enriched PV40-correlated signatures were most frequently represented by neuron and glial cell markers, along with endothelial cells, demonstrating the concomitant maturation of neurons and oligodendrocytes with lung maturation.

To determine the prognostic utility of our identified gestation-associated gene signatures, we developed a principal component–based scoring method, leveraging tissue-specific genes that differentiate term and PTC samples (Figure 4D). Specifically, this method considers RNA-based maturity as a function of the first principal component–associated variance from a PCA analysis limited to term and preterm samples. The associated gene loadings were then used to project the antenatal corticosteroid-treated samples into the established PCA space using the predict function in the software PCAtools (Figure 4E). Comparison of lung maturity using the lung RNA maturity model (RMM) found that the majority of antenatal corticosteroid–treated animals (27 of 36) had a higher predicted maturity than the upper quartile of PTCs, with a Spearman’s (partial, year) correlation of 0.35 (*P =* 0.023) between the RMM and PV40. Similar to the PV40 data, the lung RMM predicted higher doses of i.m. Celestone (1:1 mixture of betamethasone-acetate and betamethasone-phosphate) to have the most significant lung maturation score (Dunnett’s contrast *P* < 0.001) (Supplemental Table 14). Application of the independent amniotic fluid RMM to the treatment cohort produced results consistent with the lung RMM (Spearman’s partial [year] ρ = 0.45; *P =* 0.008) and found that both lung and amniotic fluid RMM predicted Celestone and i.m. betamethasone-acetate treatments to result in the greatest maturation benefit (Dunnett’s contrast *P* < 0.02), with lower predictions for both oral antenatal corticosteroid regimens (per os [p.o.] betamethasone-phosphate and p.o. dexamethasone-phosphate), mirroring the physiological effects of these treatments and doses. When the independent hippocampus RMM (based on prior samples from PsychENCODE) was applied to all hippocampus samples, we did not find any treatments that result in improvement in maturity (Dunnett’s contrast *P* < 0.05), but we observed accurate maturity predictions for our independent preterm and term controls, suggesting that antenatal steroid–induced fetal lung maturation is not associated with brain maturity. Finally, to determine if the lung RMM was applicable to amniotic fluid samples, we applied the lung model to all amniotic fluid samples, including term and preterm. While it was not inherent that such a model would correctly assign term and preterm samples as the most mature and immature samples, respectively, we did indeed obtain this result (Figure 4E and Supplemental Figure 6). While we observed an improved maturation prediction by i.m. betamethasone-acetate (B060 group) in the amniotic fluid versus lung, we attribute this effect to the broader transcriptomic impact of this treatment in the amniotic fluid, resulting in a greater number of term-induced genes (Supplemental Table 6). Furthermore, while all preterm samples displayed a more compressed dynamic range, more similar to each other, both Celestone and i.m. betamethasone-acetate protocols were again predicted to provide the greatest benefit, further suggesting that RNAs present in both lung and amniotic fluid can serve as precision markers of both normal and corticosteroid-induced lung function and organ maturation.

### Target and side effects of antenatal corticosteroids on amniotic fluid, lung, and hippocampus transcriptome.

**Figure 5:**
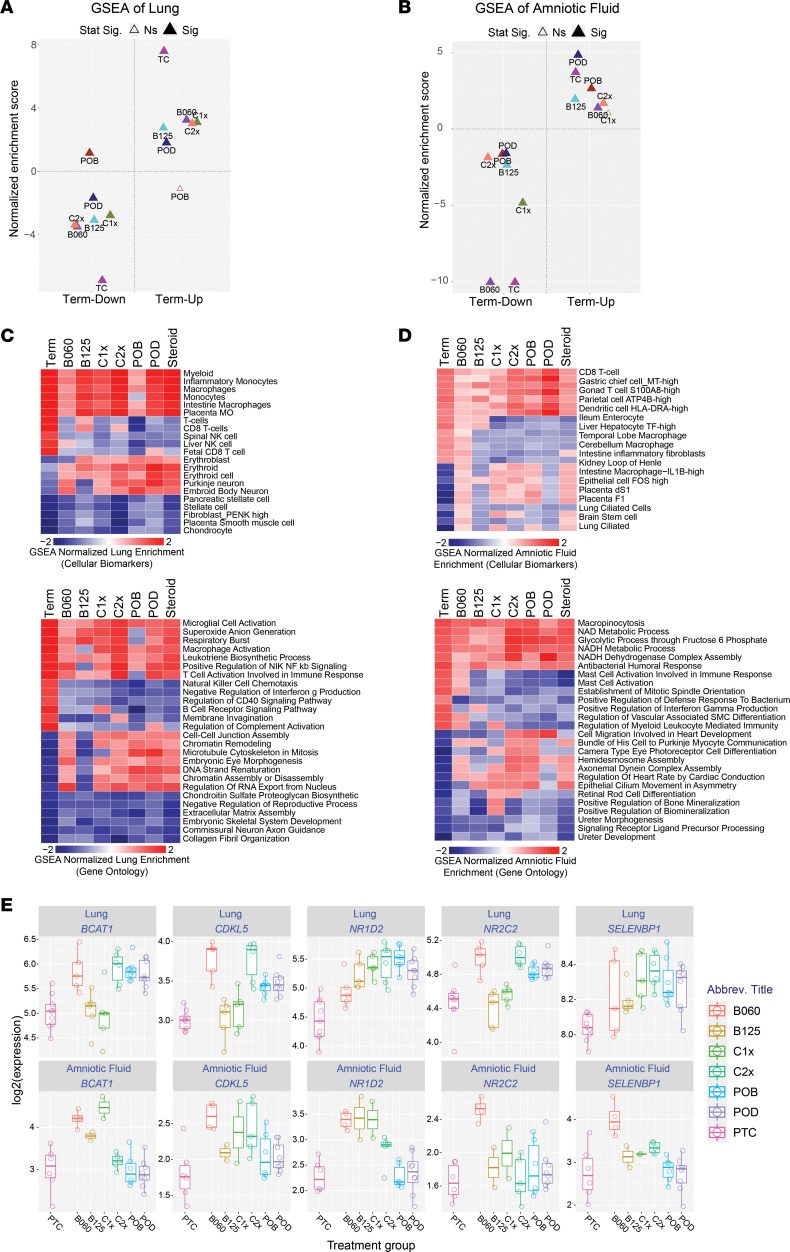
Treatment-specific and common pathways impacted in rhesus lung and amniotic fluid. (**A** and **B**) Comparison of gene set enrichment results for each steroid treatment regimen in (**A**) lungs or (**B**) amniotic fluid relative to term versus preterm impacted genes (up- or downregulated), to identify consistent maturation impacts. Top 500 up- and downregulated genes for each signature were used for GSEA. The filled triangulates indicate *P <* 0.1 (FDR corrected). (**C** and **D**) Heatmaps of gene set enrichments for each steroid treatment regimen or term versus preterm controls, to clarify maturation, common treatment or specific regimen impacts for GO terms or cellular biomarkers (AltAnalyze). (**C**) Heatmaps for lung. (**D**) Heatmaps for amniotic fluid. Full differential expression gene sets *P* value ordered (signed according to the fold direction) are provided for all fgsea analyses. Heatmap color coding uses fgsea normalized enrichment score, with red indicating positive and blue indicating negative enrichment. *n* = 47 animals for lung and 35 animals for amniotic fluid. (**E**) Box plots common upregulated genes in fetal lung and amniotic fluid comparing corticosteroid treated versus preterm controls. Open circles denote individual biological replicates lung (FC ≥ 1.5, unadjusted *P* < 0.05) and amniotic fluid (log[FC] > 0, unadjusted *P* < 0.05).

Antenatal steroids are expected to induce maturation-specific responses, as well as off-target effects, particularly in the brain. Independent of our RMM, we compared treatment-specific differential expression *P* values for all genes (with their signed direction of regulation — positive or negative) to find treatments with the greatest positive or negative similarity to term-regulated transcripts. In the lung and amniotic fluid, gene set enrichment analysis (GSEA) found evidence of concordant maturation predictions between up- and downregulated genes for all treatments but lungs from p.o. betamethasone-phosphate–treated fetuses (Figure 5, A and B). 

In lung, we found genes associated with respiratory burst, leukotriene biosynthesis, NF-κB signaling, and macrophage and T cell activation to be the most frequently shared enriched (GO) terms in term and treatment comparisons (Figure 5C). Conversely, genes specifically enriched in term versus preterm, and not steroids, were enriched in NK cell chemotaxis, negative regulation of IFN-γ, and regulation of CD40 signaling. Genes specific to antenatal steroid treatment included TGF-β signaling, chromatin remodeling, splicing, and cell-cell junction assembly. In amniotic fluid, genes most specific to steroid treatment were associated with cardiac contraction, ciliated lung cells, and brain stem cells, suggesting both cardiac- and brain-specific effects in addition the lung (Figure 5D). Many of these genes — in particular, the steroid response genes (*NR1D2*, *NR2C2*) — show a generally common pattern of upregulation in lung and amniotic fluid with corticosteroid treatment when binning samples according to treatment group (Figure 5E). In general, certain corticosteroid regimens were more consistently regulated in lung and amniotic fluid (betamethasone and single-dose Celestone 1×) versus others (Celestone 2× and oral treatments).

**Figure 6:**
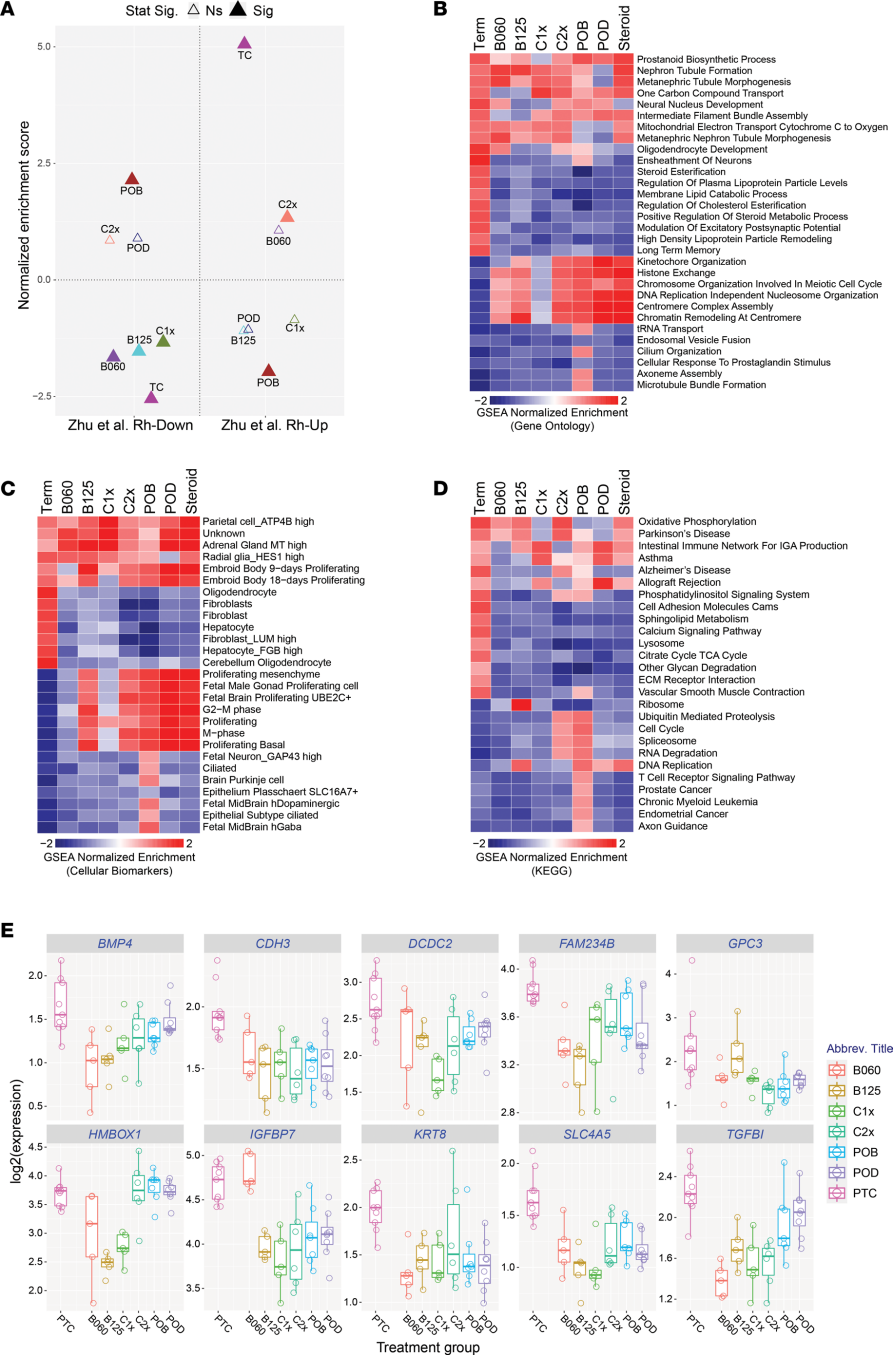
Steroids alter neuronal developmental pathways in vivo. (**A**) Comparison of gene set enrichment results for each steroid treatment regimen in hippocampus relative to term versus preterm impacted genes (up- or downregulated), to identify consistent maturation impacts. Top 500 up- and downregulated genes for each signature were used for GSEA. The filled triangulates indicate *P <* 0.1 (FDR corrected). (**B**–**D**) Heatmaps of gene set enrichments in the hippocampus for each steroid treatment regimen or term versus preterm controls, to clarify maturation, common treatment, or specific regimen impacts for (**B**) GO terms, (**C**) single-cell biomarkers, or (**D**) curated pathways. Full differential expression gene sets *P* value ordered (signed according to the fold direction) are provided for all GSEA analyses. Heatmap color coding uses GSEA normalized enrichment score, with red indicating positive and blue indicating negative enrichment. *n* = 47 animals for lung, 35 animals for amniotic fluid, and 46 animals for hippocampus. (**E**) Gene expression box plots for selected genes illustrating the range of intratreatment variation among hippocampal transcripts induced by the different steroid regimens. Selected genes are from the set of *n* = 38 genes with log_2_ fold change < –0.4 and an unadjusted *P* < 0.05 in the comparison of combined steroid treatment samples to preterm controls.

In the hippocampus, we observed poor concordance of maturation predictions compared with lung and amniotic fluid, indicating again that steroid treatment does not enhance brain maturation (Figure 6A). Given these results, we sought to define the predominant impacted genes and pathways for each antenatal corticosteroid treatment or for all treatments combined into a single group. Considering all treatment groups combined, we observe 163 differentially expressed genes (fold > 1.2 and *P <* 0.05, FDR corrected) versus only 55 genes in lung for same comparison and thresholds (Supplemental Table 15). Most of these steroid-impacted genes (74%) were downregulated and associated principally with extracellular remodeling (*COL4A6*, *MXRA5*, *TGFBI*, *POSTN*, *COLEC12*, *FBLN1*, *IGFBP7*, *COL21A1*, *TNR*), BMP signaling (*BMP4*, *SMAD3*, *SFRP1*, *SULF1*, *ROR2*, *GPC3*, *COL2A1*, *CRIM1*, and *SOSTDC1*), WNT signaling (*FZD6*, *IGFBP2*, *TCF7*, *TMEM88*, *DCDC2*, and *CDH3*), fetal ciliated-epithelial glial (ependymal) cells (*ENPP2*, *GALNT11*, *HTR2C*, *SLC16A10*, *SLC17A8*, *SLC4A5*, *TPD52L1*, *TRPM3*, *VAT1L*, and *WFIKKN2*), and glial differentiation (*ABL2*, *ENPP2*, *NTRK2*, *NDRG1*, *PTPRJ*, and *SLC45A3*) (Figure 6B). WNT signaling is a master regulator of neurogenesis and neuronal differentiation (27), while BMP4 has been shown to increase GFAP^+^ astrocytes at the expense of self-renewing GFAP^+^ neuronal progenitors (28). These findings suggest that antenatal steroids disrupt normal neurogenesis and neuronal differentiation at a critical stage of brain development. Analysis using the MSig database identified transcriptional targets (conserved cis-regulatory motif enriched) of TCF3 (E2A immunoglobulin enhancer binding factors E12/E47) as the most highly enriched transcriptional regulator (*P =* 3 × 10^–3^, FDR corrected) and genes with high CpG-density promoters bearing histone H3 dimethylation at K4 and trimethylation at K27 in the brain as the most highly enriched signature (*P =* 2 × 10^–6^, FDR corrected) (29). When considering each individual steroid treatment and term-induced genes, relative to PTCs, we further identify patterns shared among these conditions and those unique to each. Common enriched gene sets among the different treatment groups include prostanoid biosynthetic process and radial glial cell programs, while genes unique to term related to steroid esterification, lipid metabolism, oligodendrocytes, ensheathment of neurons, and long-term memory (Figure 6, C and D). Conversely, cell proliferation represented the strongest common shared pathways, unique to different steroid treatments. In many cases, these individual genes (e.g., *BMP4*, *CDH3*, *SLC4A5*, *KRT8*) were consistently downregulated with different corticosteroid regimens compared with PTCs, while for others genes (e.g., *FAM234B*, *HMBOX1*, *TGFBI* in nonoral doses), the effects were highly specific or more pronounced with certain treatments (Figure 6E). Hence, we identified gene networks impinging upon synaptic transmission and neuronal and glial maturation that are dysregulated with antenatal corticosteroid treatment that could have important clinical significance to infants exposed to these drugs.

## Discussion

Fetal maturity testing has been limited by the lack of accuracy and markers for global fetal assessment beyond the lung, although it is still used by most obstetricians and maternal-fetal medicine specialists (30). Here, we provide proof of concept that amniotic fluid contains fetal maturity markers indicating normal gestational advancement, as well as distinct changes due to corticosteroid treatment. With advancement of gestational age from 80% to near-term, we found that there are significant changes in the amniotic fluid transcriptome that correlate with transcriptomic changes in the fetal lung and the fetal hippocampus in rhesus macaques. In addition, the transcriptomic effects of antenatal corticosteroid treatment on the lung and hippocampus overlap with differences in the amniotic fluid transcriptome. These findings have important implications for future research and clinical practice. First, we can use screening of the amniotic fluid, which has a larger proportion of cell-free fetal RNA and DNA than maternal blood, to detect organ-specific fetal maturation biomarkers. The eventual goal will be to develop a less-invasive test on maternal serum to search for these identified biomarkers, which in combination with other studies, such as imaging, could provide a more comprehensive assessment of fetal maturity (31). In addition, we can use transcriptomic changes in the amniotic fluid to detect tissue-specific effects of antenatal exposures, such as corticosteroids. These findings could lead to new methods for detecting effects of antenatal exposures in humans and guiding the delivery of complicated pregnancies.

Obtaining tissue-specific information on fetal maturation could allow for personalized prenatal and postnatal care of preterm infants. Such individual-specific information could tailor the use of antenatal therapies, such as antenatal corticosteroids or magnesium, to improve neonatal outcomes and improve the ability to detect patients that would benefit from other prenatal interventions in future trials. Moreover, as our understanding on the dosing of antenatal corticosteroids improves, it could provide additional guidance on the formulation, dosing, and length of therapy (32, 33). It could also help detect specific pathways of beneficial and detrimental effects of antenatal corticosteroids to the fetus and identify surrogate markers for use in clinical trials for new indications of this therapy, such as late preterm deliveries or prior to elective cesarean. Based on the results from our in silico predictive model, amniotic fluid generally represents a proxy for the prediction of lung maturation. However, we note greater gene expression impacts for some treatments in the amniotic fluid, compared with the lung, suggesting possible gene expression impacts in nonlung tissue that may contribute to these scores. As such, these molecular differences may not directly relate to maturity, lung compliance, or other clinical outcomes.

We have focused on the fetal lung and hippocampus because of the relevance those organs have for short- and long-term outcomes of preterm infants. Historically, lung immaturity has been a major obstacle to the survival of preterm infants, and even today, pulmonary complications continue to be the most common morbidity among preterm infants (34, 35). As the survival of preterm infants has improved over the last several decades, focus has shifted to the improvement of neurodevelopmental outcomes of extremely preterm infants (36, 37). Despite the established beneficial effects of antenatal corticosteroids in improving the survival of these infants, concerns regarding their safety remain due to the unknown effects on long-term neurodevelopmental outcomes, especially among late preterm pregnancies and the 40% of corticosteroid-treated pregnancies that do not deliver preterm but unnecessarily received the intervention (32, 38, 39). Population-based studies have shown that term-born infants exposed to antenatal corticosteroids are at increased risk of behavioral and neurodevelopmental problems (38, 40), highlighting the need for a more targeted use of antenatal corticosteroids. During fetal life, the hippocampus is rich in glucocorticoid receptors, and concerns exist about the detrimental effects of antenatal corticosteroids to brain development. Specifically, increased apoptosis in the hippocampus of rhesus macaques was noted (41), which is consistent with findings of decreased neuronal density of human newborns and suppressed neuronal development signaling in rhesus macaques exposed to antenatal corticosteroids (9, 42). We found that antenatal corticosteroids modulate important brain developmental networks with suppression of signaling pathways that regulate neurogenesis and neuronal differentiation. Moreover, corticosteroid-treated fetuses did not display transcriptional signals of improved brain maturation.

It is particularly interesting that different dosing regimens of the same corticosteroids had different effects on the fetal transcriptome. Glucocorticoids exert their effects by binding to the glucocorticoid receptor in the cytoplasm, leading to both genomic and nongenomic effects via protein-protein interactions. The genomic effects of the glucocorticoid receptor are complex, with multiple mechanisms of gene transcriptional induction and repression (43). It has also been postulated that glucocorticoids may have nongenomic effects independent of binding to the glucocorticoid receptor (44). In lung epithelial cells, the effect of corticosteroids on specific genes was dependent on dose (45). Corticosteroids can also increase accessibility to DNA regions that are not normally accessible; thus, an initial dose of corticosteroid can modulate the response to subsequent doses (46). Physiologically, we have noted that the initial peak provided by the betamethasone phosphate component decreases the efficacy of the therapy in improving lung compliance while impairing fetal growth (47). The complexity of mechanisms of action with varying binding affinities may contribute to the diversity in response seen in our study, since the availability of the corticosteroid, of the glucocorticoid receptor, and of DNA binding sites would influence which of these mechanisms are activated. It is also important to note that the corticosteroid treatment did not result in a term-equivalent transcriptome, suggesting that treatment with corticosteroids also induces changes not related to maturity that need to be further explored.

One of the limitations of this study is the use of nominal *P* values instead of adjusted *P* values in our differential expression analyses, but the use of effect-size cut-offs, validation with single-cell data sets, and focus on overlaps and GSEA from these genes provided additional layers of screening for identifying biologically relevant results. In addition, the timing of tissue collection on animals treated with antenatal corticosteroids influenced the results and identification of corticosteroid-related changes in the transcriptome. There is a disconnect between the timing of peak RNA changes induced by antenatal corticosteroids, which happens early after administration of the drugs, and the timing of measurable fetal lung maturation, which happens after the RNA signaling has decreased (9). Hence, it is possible that, if measured early after administration of antenatal corticosteroids, the overlap of differentially expressed genes between fetal tissue and the amniotic fluid would be greater than what we reported, but we would be unable to accurately report fetal lung maturation induced by the treatment. The time point at 5 days after injections allows clear identification of lung compliance changes induced by corticosteroids. Another limitation is the number of samples available — particularly for the hippocampus from term fetuses. We used previously published scRNA-Seq and bulk RNA-Seq data sets from macaques and bulk RNA-Seq data sets from humans at different stages of development to validate our findings in the hippocampus. While enrichment for genes associated with vasculature in the brain and amniotic fluid is not specific to the brain, it may reflect the rapid growth the brain is undergoing at this time in gestation, which is followed by growth of the brain vascular system. More importantly, overlap of genes differentially regulated in the hippocampus and amniotic fluid revealed genes associated with oligodendrocyte maturation and myelin sheath formation (*GSN*, *MAL*, *MT3*, and *CSRP1*), suggesting that gene changes associated with myelination are reflected in the amniotic fluid. The lack of maturation signature in the hippocampus of corticosteroid-treated fetuses and suppression of neurogenesis regulators is of particular importance for future studies of antenatal corticosteroids to include neurodevelopmental outcomes as an endpoint.

Overall, we found that cell-free fetal RNA in the amniotic fluid correlates with tissue-specific transcriptomic changes and provides a platform for discovery of fetal maturation and health markers. These could include tissue-specific splice isoforms, in which this compendium of matched-tissue RNA-Seq may be useful for future studies. We previously reported such differences in splicing isoform usage, comparing human term and preterm amniotic samples, as well as those with and without clinical complications, against adult tissue profiles (18). Indeed, we detect hundreds to thousands of shared splicing events in amniotic fluid, uniquely common to our profiled lung or brain samples (Supplemental Figure 7). While our results are a proof of concept that the amniotic fluid can provide information of fetal maturation and prenatal exposures, in the clinical setting, the ideal method would consist of a less-invasive, targeted testing that can provide quick-turnaround results. Our findings need to be further corroborated in human cohorts — not only at different gestational ages, but also after fetal treatments — for similarities in gene expression patterns in the amniotic fluid and correlation with clinical outcomes of newborns. Further investigation of cell-free RNA in the maternal serum could provide a less-invasive, alternative test for fetal maturation and well being.

## Methods

### Animals.

Time-mated pregnant rhesus macaques were delivered preterm at 131 days of gestation or near term at 155 days of gestation (full term is 165 days). For antenatal corticosteroid dosing studies, preterm pregnant rhesus macaques received one of the treatments listed in Supplemental Table 1. Oral drugs were given to animals habituated to receiving small treats, as previously reported (9, 48). The i.m. betamethasone-acetate 0.125 mg/kg and the oral betamethasone-phosphate have been previously shown to induce fetal lung maturation similar to the standard clinical treatment in preterm rhesus macaques, while the betamethasone-acetate 0.06 mg/kg and the oral dexamethasone-phosphate did not (9, 48).

Fetuses were delivered 5 days after the initiation of treatment with antenatal corticosteroids at 131 ± 5 days of gestation by cesarean section with intact membranes (*n =* 5–8 animals/group). After delivery, the amniotic fluid was sampled and immediately stored in AssayAssure tubes with standardized buffer (Sierra Molecular Corporation) and frozen at –80°C. After euthanasia, pressure-volume curves were measured by inflating lungs to 40 cmH_2_O, followed by deflation, with measurements of lung volumes performed using a syringe and pressure manometer. Samples from the right lower lobe of fetal lung and the hippocampus were snap frozen for RNA-Seq.

### RNA isolation and sequencing.

Approximately 10 mL of amniotic fluid from each animal was used for cell-free RNA extraction with the QIAamp Circulating Nucleic Acid Kit (QIAGEN). RNA from the lung and hippocampus was extracted with the RNeasy Universal Mini Kit (QIAGEN). The RNA-Seq libraries were prepared using the TruSeq RNA Library Prep Kit v2 (Illumina), with paired-end 75 bp RNA-Seq performed with a HiSeq 2500 (Illumina). Samples were sequenced at a target depth of 30 million reads by the Cincinnati Children’s Hospital Sequencing Core. The sequencing data has been deposited in GEO (GSE171669; https://www.ncbi.nlm.nih.gov/geo/query/acc.cgi?acc=GSE171669).

### RNA-Seq analysis quantification.

We combined the raw RNA-Seq data from the current study (*n =* 110) with lung and brain samples sequenced in our prior study (*n =* 18, PTC and antenatal steroids [GSE118438]), using the identical experimental protocols (9, 48) (Supplemental Table 1). To quantify gene expression, we applied pseudoalignment with the software Kallisto using the Ensembl 91 Rhesus Macaque reference transcriptome. Transcript level information was converted to gene level TPMs using the software AltAnalyze version 2.1.4. Data from one amniotic fluid sample was not used because the distribution of expression values for this sample was markedly lower than for the other samples. For downstream analyses, rhesus gene symbols were converted to human gene symbols, where possible (Ensembl BioMart). All PCA were conducted using the PCA function supplied in the PCAtools R package, with the center and scale options set to “TRUE”. The number of PCs to retain for covariate associations was determined using the Elbow method and displayed in scree plots (Figure 1B and Supplemental Figure 1, A–C).

To augment and validate the above hippocampus term and preterm samples, we downloaded preprocessed expression results using bulk RNA-Seq from PsychENCODE — specifically, gene-level counts from 5 hippocampus samples (http://www.evolution.psychencode.org/files/processed_data/RNA-seq/). The primary samples of interest in this cohort from Zhu et al. (25) were *n =* 3 (all males) obtained at gestational age = 110 days and *n =* 2 (1 male, 1 female) obtained at birth (P2 and P0). Count data was transformed to the log2(1 + counts per 10,000 [CPTT]) scale and subjected to PCA. PCA on the 5 primary samples and the full set of 10 samples indicated a marked effect of sequencing platform. Differential expression analysis comparing the 2 newborn samples to the 3 GA110 samples was conducted using the DESeq2 R package. Specifically, the DESeq function was used to model counts as a function of platform and age group, with default options for size factor and *P* value estimation. The results function was used to apply independent filtering for multiple-test correction, with default option setting of α = 0.10 and the adjustment method of Benjamini-Hochberg (FDR) (Supplemental Table 6). Human RNA-Seq gene expression data (RPKM normalized) from neonate, infant, and preterm hippocampus were further obtained from the Allen Brain Atlas database (http://www.brainspan.org/static/download.html). Specifically, *n =* 3 preterm samples at 21 and 24 weeks after conception (postweek conception [pwc]) and neonate as 37 pwc, 4 months postnatal (*n =* 2), and 1 year old (*n =* 1). Statistical analyses for these data were conducted in AltAnalyze, with upregulated (*n =* 417) and downregulated (*n =* 754) gene sets consisting of those meeting an eBayes moderated 2-tailed *t* test *P* ≤ 0.05 and fold change ≥ 2.0.

Alternative splicing and alternative promoter estimate for each RNA-Seq sample were obtained from the STAR aligned (Ensembl 91) BAM files in the software AltAnalyze version 2.1.4 (MultiPath-PSI algorithm). The splicing events for all samples are reported in Supplemental Table 16. Venn diagram visualization was performed in AltAnalyze.

### Removal of genes with sex-linked profiles.

Initial exploratory PCA plots of gene expression data indicated marked segregation between samples from male (M) and female (F) fetuses. Since the treatment groups were not balanced on M/F ratio, we sought to remove sex effects in gene expression due to inherent M/F differences (e.g., on Y chromosome) while retaining differences that may be related to fetal development in the context of this research. First, genes with average log_2_(TPM) values ≤ 0.1 across samples in each tissue were removed from consideration to limit the identification of sex-related genes to those with reliable expression information. We then formed separate datasets for the 2017 and 2018 samples for each tissue. PTC samples were removed due to inadequate M/F representation. This process resulted in the 6 data sets having adequate numbers of samples to estimate sex effects while controlling for treatment group. The limma R package (voom variance adjustment) was used to estimate the M/F difference in gene expression, while controlling for treatment group, in each data set. Genes with consistent effects (direction and raw *P* ≤ 0.05) across years within a tissue were considered candidates for removal. Examination of log fold change distributions was used to refine the list of genes to remove — specifically, we applied the requirement of consistent log_2_ fold changes of at least 0.25 or –0.15. Finally, we aggregated information across the 3 sample types and compared genes identified to a recently published blood transcriptome analysis (including sex differences) of breast-feeding rhesus macaque infants (49). A total of 17 genes were, thus, identified and removed from each expression matrix (Supplemental Table 2). These 17 genes represent 0.08% of the 21,385 genes quantified and a maximum of 0.23% of the total of counts for each sample.

In the analysis of the macaque hippocampus data from Zhu et al. (25), the differential expression analysis identified a number of upregulated X-linked genes and downregulated Y-linked genes with marked differences, presumably due to 1 female sample in the newborn group and none in the comparator group. Specifically, 10 of the 17 genes identified above (*DDX3Y*, *USP9Y*, *UTY*, *ZFY*, *KDM5D*, *EIF1AY*, *TBL1Y*, *NLGN4Y*, *DDX3X*, *KDM6A*) and an additional 4 X-linked genes (*XIST*, *Xist_exon4*, *JPX*, *FTX*) were removed from the set of differentially expressed genes.

### Batch-effects correction.

To correct for temporal differences in RNA collection and processing, we performed batch-effects correction using NOISeq package in R, after excluding genes with strong sex-linked profiles (*n =* 17). NOISeq was run using the ARSyNseq correction function, specifying the treatment group variable rather than a specific set of batch effect factors due to the number and interrelatedness of candidate batch-effect factors. After batch-effects correction, gene filtering was done based on mean log_2_ expression values across samples, separately for lung, brain, and amniotic fluid. Genes were retained for differential expression, and correlation analyses if mean log_2_ expression values (prior to batch correction) were ≥ 1.0 TPM. Use of unfiltered data in some analyses (e.g., some deconvolution analyses) is noted in the analysis descriptions.

### PV40 by treatment group.

PV40 measurements were displayed in a box plot format, broken out by treatment group and with sample sex indicated (Figure 4A). Two PTC samples with implausibly high PV40 values for a preterm fetus were omitted from this analysis. ANOVA of PV40 values by treatment group (1-way), with and without a sex covariate (2-way), were conducted using the R function aov. Accompanying residual diagnostics for homogeneity of variance (Levene’s test) and normality (Q-Q plots) indicated model assumptions were sufficiently satisfied. The overall *P* value for treatment differences in each analysis was < 0.05; thus, each treatment was compared with the PTC samples using Dunnett’s test (Supplemental Table 11).

### RNA-Seq correlation analyses.

Correlation of gene expression with PV40 measurements was quantified, by sample type, by Spearman’s partial correlation coefficient, with sample year (dichotomized to 2018 versus other) as the control variable. Two PTC samples with implausibly high PV40 values for a preterm fetus were omitted from this analysis. Computations, including calculation of *P* values, were done using the pcor.test supplied in the ppcor R package (Supplemental Table 12). Gene set enrichment of genes correlated with PV40 (positive, raw *P* < 0.05) was conducted for each sample type using the software GO-Elite using a large collection of prior reported single-cell, cell type markers (Figure 4, B and C, and Supplemental Table 13).

Correlations of gene expression between lung and amniotic fluid sample pairs and hippocampus and amniotic fluid sample pairs were computed using the same methods described above. A total of 35 animals had both lung and amniotic fluid samples; 34 had both brain and amniotic fluid samples. After applying the gene filtering described above and merging of the sample data, correlations were calculated for a total of 7,380 genes in the lung/amniotic fluid analysis and 7,198 genes in the brain/amniotic fluid analysis. Results were displayed in volcano plots with coloring and labeling of points done based on a *P* value (unadjusted *P* *≤* 0.05) criterion (Figure 3 and Supplemental Table 9). Gene set enrichment of lung/amniotic fluid– and brain/amniotic fluid–correlated genes (positive, raw *P* < 0.05) was conducted as described above (Figure 3 and Supplemental Table 10). Gene network diagrams appearing in Supplemental Figure 5 were produced using the software GO-Elite. Each enriched gene set (central square node) is connected to transcripts (red-colored dots) that are positively correlated in the lung and amniotic fluid samples from the same animals. Blue-colored nodes in panel A denote tissue or cellular markers (AltAnalyze TissueMarker database) and yellow-colored nodes in panel B denote putative regulatory transcription factors. For predicted transcription factor targets, interactions from the PAZAR and Amadeus databases was used (GO-Elite). For tissue-specific genes, the AltAnalyze TissueMarker database was used, with markers defined using the MarkerFinder algorithm applied to diverse tissues and purified cell-types (18) (Supplemental Figure 5).

### Differential gene expression.

Differential gene expression was evaluated using the limma R package applied to the batch-corrected log_2_(1+TPM) expression values and was done separately for each sample type. The model incorporated blocking of treatments within sample year (dichotomized to 2018 versus other) to correspond to the 2 main treatment-by-year subsets. This ensured treatments were compared with (reasonably) contemporary PTCs and provided additional control for any year effects not removed in prior steps. The trend=TRUE and robust=TRUE variance estimation parameters were used in the call to the limma eBayes function (Supplemental Table 6). Contrasts were estimated for each steroid treatment versus the PTCs. The Benjamini-Hochberg FDR correction was applied to the aggregate collection of *P* values from all contrasts, separately for each sample type. DEGs from the limma analyses described above, meeting raw *P* ≤ 0.05 and absolute value of log_2_ fold change ≥ log_2_(1.25) were used in GSEA and displayed volcano (Figure 2D and Supplemental Figure 4A) and UpSet-style plots (Figure 2E). Note that UpSet-style plot marginal totals for each intersection are displayed rather than the number of DEGs unique to each particular intersection. Output from the R package UpSetR (UpSet function) was used to make the bottom of the plot, and the R package ggplot2 was used to create the vertical bars with counts on the top portion of the plot. For selection of individual genes to assess variation among biological groups, we applied a log_2_ fold change < –0.4 with an unadjusted *P <* 0.05 to identify 38 genes in hippocampus corticosteroid treated versus PTCs, of which 10 examples are displayed. To select genes with common corticosteroid impacts in lung, we select 6 genes upregulated with a fold change > 1.5 and an unadjusted *P <* 0.05 in lung and an unadjusted *P <* 0.05 with any positive increase (aggregate corticosteroid treated versus PTCs).

### GSEA.

Indicated GSEA were performed in the software GO-Elite, through AltAnalyze (50, 51). Prior to enrichment, separate gene lists were created for positive and negative log fold changes/correlations with filtering criteria of unadjusted *P* ≤ 0.05 and additionally abs(log_2_[fold change]) ≥ log_2_(1.25), where abs represents absolute value, for results from DEG analyses. The MarkerFinder algorithm was used to provide additional treatment group-based marker gene sets with GO-Elite enrichment results, including heatmaps from AltAnalyze (Figure 1C and Figure 2).

Comparisons of steroid treatment effects to term versus preterm differences were conducted using GSEA as implemented in the R package fgsea (52). To do so, the DEG analysis (as described above) results vector for each steroid treatment was sorted in decreasing order of the *t* test statistic. The term versus PTCs gene sets for the lung and amniotic fluid assessments consisted of genes for which the unadjusted *P* value was ≤ 0.05 and the log_2_(fold change) was ≥ log_2_(1.25) (upregulated set) or ≤ –log_2_(1.25) (downregulated set) in the differential gene expression analysis (Figure 5A and Figure 6, A and B). The hippocampus term versus preterm gene sets consisted of genes identified in the DEG analysis of the Zhu et al. (25) samples, subsetted to those genes present in the results vectors. Normalized enrichment scores (enrichment score divided by mean enrichment of random samples of the same size) and *P* values were computed with the values of the eps and nPermSimple parameters adjusted as needed to achieve sufficient precision of *P* value estimates. Results from DEG analyses, including combined steroid samples compared with PTCs, were also examined for enrichment in gene sets from GO and KEGG pathway databases and a large collection of prior reported single-cell, cell type markers (Ensembl-BioMarkers.txt) (Figure 5, B–D, and Figure 6, C and D). The KEGG gene sets (human) were obtained using the Bioconductor package KEGGREST; GO gene sets were obtained using the Bioconductor packages GO.db and org.Hs.eg.db. Only gene sets with ≥ 3 genes in the DEG results were evaluated.

### RMM.

A maturation measure was developed for each sample type by applying the following steps: (a) PCA reduction was estimated for a reduced data set limited to PTC and term control samples and genes that satisfied the following criteria applied to the results of the DEG analyses described above: lung, abs(log_2_[fold change]) ≥ log_2_(2-fold change) and FDR-adjusted *P* ≤ 0.05; amniotic fluid, abs(log_2_[fold change]) ≥ log_2_(1.5) and FDR-adjusted *P* ≤ 0.05. Zhu et al. (25) data were used for hippocampus to identify DEGs meeting FDR ≤ 0.10. (b) Data from all samples were centered and scaled (by gene) and scored on the first PC from step (a) using the PCATools predict function. (c) Sample scores were displayed by treatment in a box plot graphic. To determine the extent to which lung and brain maturation might be reflected in the amniotic fluid samples, the lung and brain measures were also used to score the amniotic fluid data. Amniotic fluid sample scores were calculated and displayed following steps (b) and (c) above using batch-corrected but unfiltered expression values (Figure 4, D and E). The lung maturation scale values (as applied to lung data) were plotted (ggplot) versus available PV40 values. A LOESS smooth function (red dashed line) reflecting the association was fit and displayed (ggplot smooth function). The Spearman’s (rank) correlation coefficient, adjusted for sample year as described above, was calculated with accompanying *P* value using the R package ppcor (pcor.test function) (Supplemental Figure 6). To test for differences in the RNA Maturity Metric between each treatment or term controls versus PTCs, we performed 1-way ANOVA followed by a Dunnett’s test to obtain a t-statistic and associated *P* value for each pairwise comparison (Supplemental Table 14). The associated R scripts for the RNA Maturity Metric is available at: https://github.com/DanSchnell/DS_Bioinformatics/tree/Rh_manuscript, commit 30edb909b3f7404b2da406c2ba24d67cb8c8d327.

### Bulk RNA-Seq deconvolution analysis.

To estimate the cellular composition of the lung, brain, and amniotic fluid samples, we applied the DeconRNASeq R package, which uses nonnegative quadratic programming to assess the relative contribution of different cell types to a bulk sample, against established single-cell populations or bulk RNA-Seq tissue compendium samples sets. Prior to analysis, we downloaded the already-processed sparse-matrix counts for a recently published human lung single-nucleus RNA-Seq data set spanning neonates through adults (22) from LungMAP (GSE161381). Gene UMI counts were scaled to the total reads per nuclei-barcode (CPTT). Subsequently, the MarkerFinder algorithm in AltAnalyze was used to identify the top 100 (unique) genes for each author annotated nuclei-population. The signature matrix entries for each population were the corresponding marker gene correlations and 0 for marker genes for other populations, as the input for deconvolution analysis. Deconvolution analysis on bulk amniotic fluid samples was also performed using tissue type signatures created using GTEx version 8 bulk RNASeq data. Gene TPM data for GTEx samples were obtained using version 8 RNASeQC (v1.1.9) preprocessed results from https://gtexportal.org, corresponding to 660 GTEx human samples representing a total of 22 broad aggregated tissue types as the reference for deconvolution. Samples from sex-specific tissue types (e.g., fallopian tube, testis), brain regions other than hippocampus, and any tissues with fewer than 5 samples, cell lines, and arteries were not included. To enable deconvolution of our rhesus amniotic fluid samples, TPM data reduced to protein coding genes (BioMart) and further filtered to only gene symbols with data in the unfiltered amniotic fluid expression data. Random subsampling was applied within each tissue type (to a maximum of 30 samples), and data were transformed using log_2_(1+TPM). MarkerFinder in AltAnalyze was used to identify the top 200 genes correlated with each tissue type. Marker genes with Pearson’s correlation > 0.5 were selected and centroids for each tissue type were computed (gene-wise means of log_2_[1+TPM]). Batch-corrected rhesus amniotic fluid TPM values and the GTEx-derived centroid matrix (converted to TPM) were supplied to DeconRNASeq using the default parameters. The results were displayed in box plot format with amniotic fluid sample markers coded by treatment group. Two of the tissue types (adipose_visceral[omentum] and spleen) had tissue RNA composition averages < 0.5% and are not among those shown. Deconvolution of the hippocampus samples was done using a reference derived from a scRNA-Seq study of the developing human fetal hippocampus (23) (Figure 2, A–C).

To establish if the estimated tissue deconvolution RNA percentage for the term control samples was the maximum (or minimum) among all treatment groups, 1-way ANOVA followed by Dunnett’s test was applied to amniotic and lung samples. Specifically, if the overall F test from the ANOVA of percentage on treatment group was significant (*P* < 0.05), Dunnett’s test (term control as the common comparator) was applied. We considered the term control percentage to be the maximum (minimum) among all treatment groups if the mean term control percentage was the numerical maximum (mininum) among all means and all the Dunnett’s-adjusted *P* values were < 0.05 (Supplemental Table 5).

We further applied a complementary *Z* score marker gene set strategy to infer the relative strength of each external tissue/cell type signature across treatment groups in our amniotic fluid, lung, and hippocampus samples (52). In short, for each tissue or single-cell population used as a reference in the deconvolution analyses, the top 20 specific markers were selected and queried in the rhesus tissue expression data, followed by normalization of the expression values in each of the rhesus samples by centering (subtraction of mean) and scaling (division by SD) of the PTC samples. These standardized values were then averaged across all the genes in a set, producing a single value for each sample for each gene set. These values were displayed by marker gene set and treatment group in box plot format (Supplemental Figure 3). To establish if the standardized gene set expression values for the term control samples was the maximum (minimum) among all treatment groups, the same 1-way ANOVA procedure used for the deconvolution data was applied (Supplemental Table 5).

### Statistics.

Statistical analyses were conducted using the R statistical software (versions 3.6.3 and 4.1.0) and AltAnalyze (version 2.1.4?); see analysis-specific sections in sections above for packages, functions, and parameter values that were used. Differential gene expression calculations were conducted using data on the log_2_(1+TPM) scale and all use batch-corrected data. All reported *P* values are 2 sided and all FDR-adjusted *P* values were calculated using the Benjamini-Hochberg method. In some cases, nominal *P* values (unadjusted, *P ≤* 0.05 or *P ≤* 0.10) were used as a filtering variable, usually in conjunction with a log(fold change) criterion, to create lists of genes for downstream GSEA. Genes for which differential expression results are specifically mentioned above satisfy a FDR-adjusted *P* ≤ 0.10 criterion. Dunnett’s test (each group compared with a common control) was used for multiplicity adjustment in both 1- and 2-way ANOVA analyses of PV40, maturity scores, and deconvolution and gene set *Z* score cell/tissue-type analyses. Box plots presented use the standard paradigm of marking data median (middle bar), first and third quartiles (outer edges of box), and whiskers that extend up to 1.5 times the interquartile range (IQR) to the furthest datum within that distance. However, all data points are plotted rather than just outliers.

### Study approval.

Pregnant rhesus macaques were studied at the California National Primate Research Center at the UCD with protocols and procedures approved by the IACUC (protocol no. 20333).

## Author contributions

Conceptualization was contributed by AFS, NS, and BDKR. Methodology was contributed by AFS, AHJ, NS, and BDKR. Formal analysis was contributed by DJS, KPE, KC, and NS. Investigation was contributed by AFS, DJS, KPE, KC, DTS, PSK, LAM, AHJ, NS, and CAC. Resources were contributed by LAM, AHJ, NS, BDKR. Data curation was contributed by DJS and NS. Writing of the original draft was contributed by AFS and NS. Review and editing of this manuscript were contributed by AFS, DJS, DTS, AHJ, NS, and BDKR. Supervision was contributed by BDKR and NS.

## Supplementary Material

Supplemental data

Supplemental table 1

Supplemental table 2

Supplemental table 3

Supplemental table 4

Supplemental table 5

Supplemental table 6

Supplemental table 7

Supplemental table 8

Supplemental table 9

Supplemental table 10

Supplemental table 11

Supplemental table 12

Supplemental table 13

Supplemental table 14

Supplemental table 15

Supplemental table 16

## Version 1 08/18/2022

In-Press Preview

## Version 2 09/22/2022

Electronic publication
