# Supplementary material for: Fetal maturation revealed by amniotic fluid cell-free transcriptome in rhesus macaques
Source: JCI Insight. 2022 Sep 22;7(18):e162101. doi: 10.1172/jci.insight.162101 (PMC9675452; doi:10.1172/jci.insight.162101)
Supplement: Supplemental data [file jciinsight-7-162101-s222.pdf]

Supplementary Figures

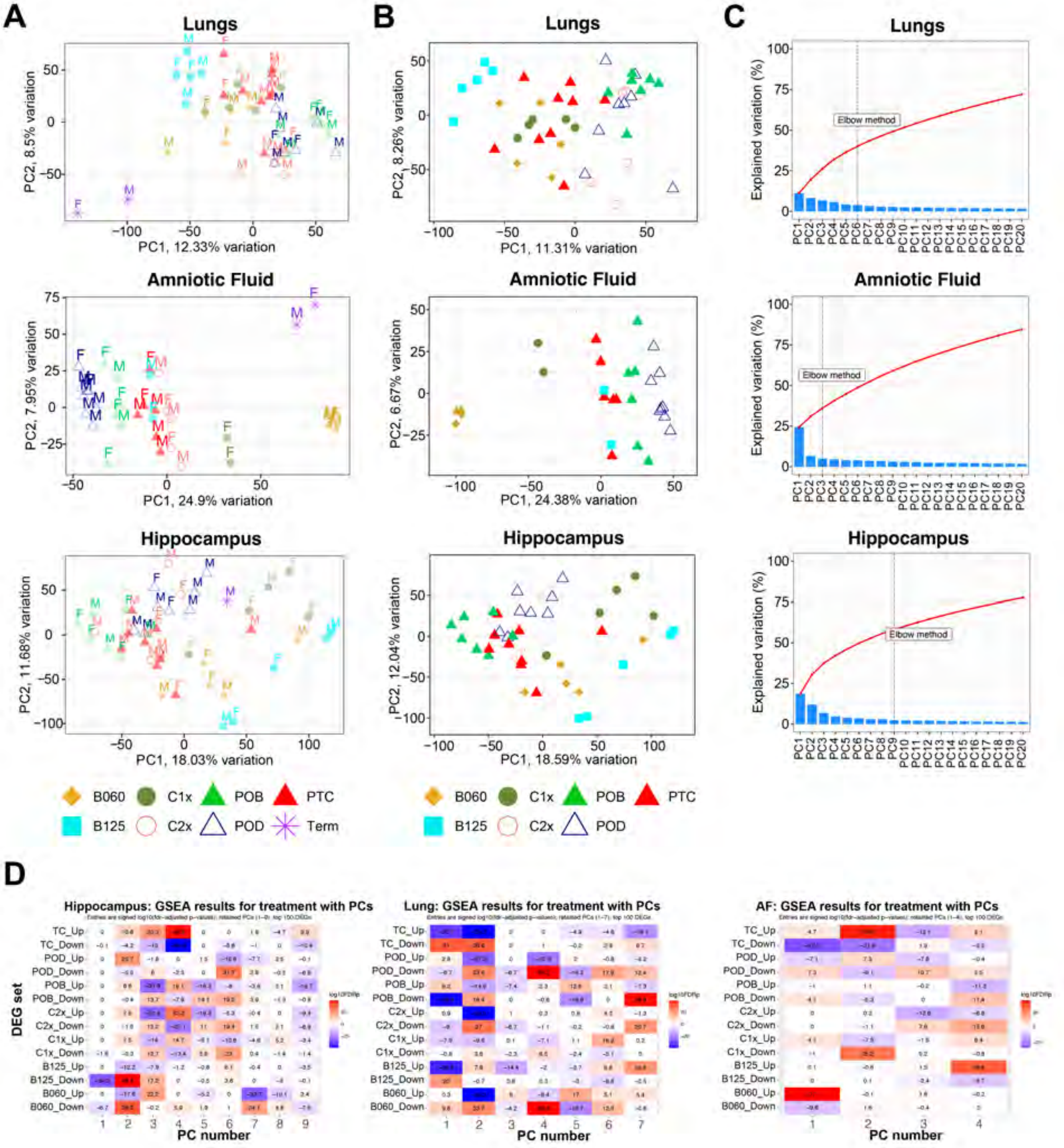

Supplementary Figure 1. Global variation in rhesus gestational tissues associated with sex and treatment. A) The first two principal components displayed for gestational samples including term samples, annotated by fetal sex (M=male, F=female). B) Same as panel A without term samples, showing a similar distribution of samples as in panel A. C) Scree plot of applying the Elbow method with Term control samples. D) GSEA of differentially expressed genes in each treatment group versus preterm controls to identify the association between estimated treatment group effects and each principal component. Differentially expressed genes are defined as limma non-adjusted p ≤ 0.05 & log2FC ≥ log2(1.25), for up- and down-regulated genes. PC gene lists were obtained by sorting (decreasing) the full set of genes used in the PC analysis by the gene loadings. The R package fgsea was used compute the

(directional) enrichment of each treatment gene set in each ordered PC gene list, i.e., the extent to which the top 100 genes in each treatment effect gene set appear at the extremes of each ordered PC gene list. The results are summarized for lung, hippocampus, and amniotic fluid data in the heatmap displays below. For each of the sample types, each of the treatment condition gene sets is associated with the set of retained PCs. These associations include PC gene lists that are significantly enriched (signed log10(FDR-adjusted p-value) >= 2, positively or negatively), with ranked differentially expressed genes for the term or treatments versus preterm controls. n = 47 animals for lung, 35 animals for amniotic fluid and 46 animals for hippocampus.

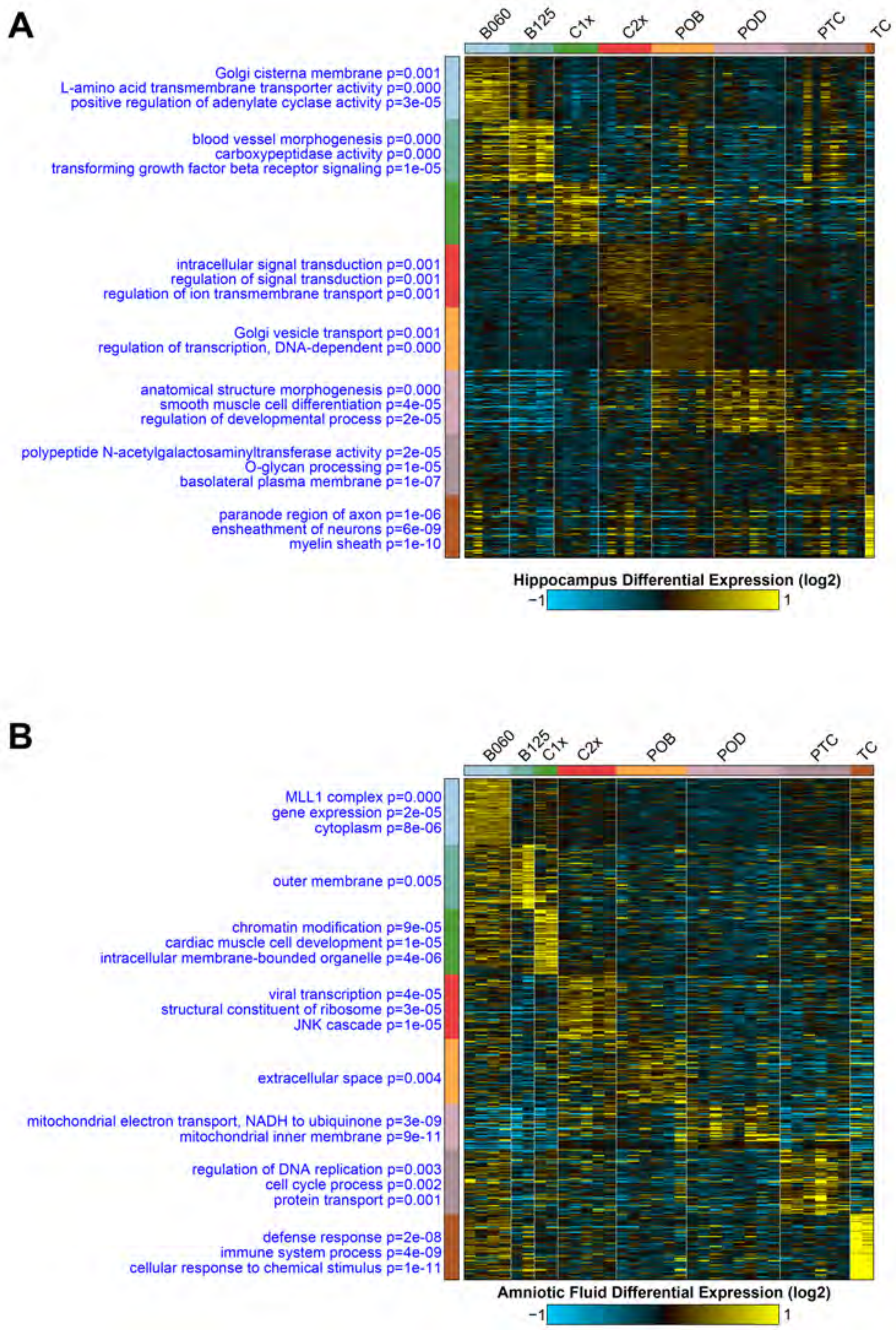

Supplementary Figure 2. Antenatal corticosteroids produce distinct transcriptional responses in fetal amniotic fluid and brain. A-B) Heatmap of the top-most specific marker genes for A) hippocampus or B) amniotic fluid RNA-Seq for each treatment. Expression values are calculated as log2 fold-changes relative to the mean of each row. The top GO-Elite Gene Ontology enrichment results are denoted to the left of each cluster along with corresponding Fischer's Exact test enrichment p-values. Gene-set enrichment results are reported for gene-sets with an enrichment z-score > 1.96, at least three genes and a Fishers Exact p<0.05 (FDR corrected). n = 35 animals for amniotic fluid and 46 animals for hippocampus.

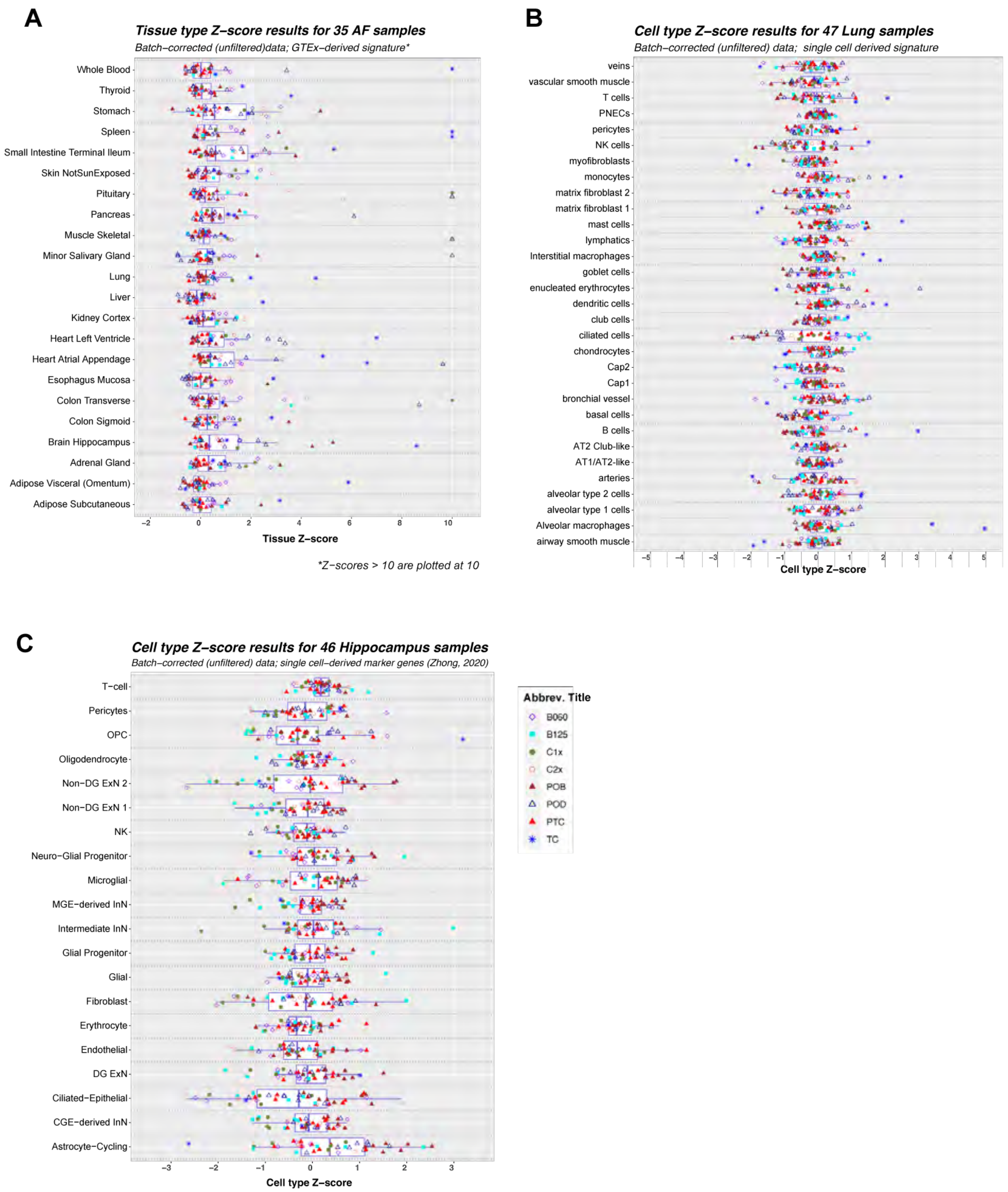

Supplementary Figure 3. Secondary evaluation of cell-type differences in fetal tissues and amniotic fluid. Secondary method using a Z-score marker gene set analysis to assess the differences in relative abundances among Rhesus fetal bulk transcriptomics tissue and amniotic fluid (Methods). A) Amniotic fluid gene-set tissue scores from human GTEx tissues. B) Fetal lung gene-set tissue scores from human infant and adult lung single-cell RNA-Seq. C) Fetal hippocampus gene-set tissue scores from human fetal hippocampus single-cell RNA-Seq.

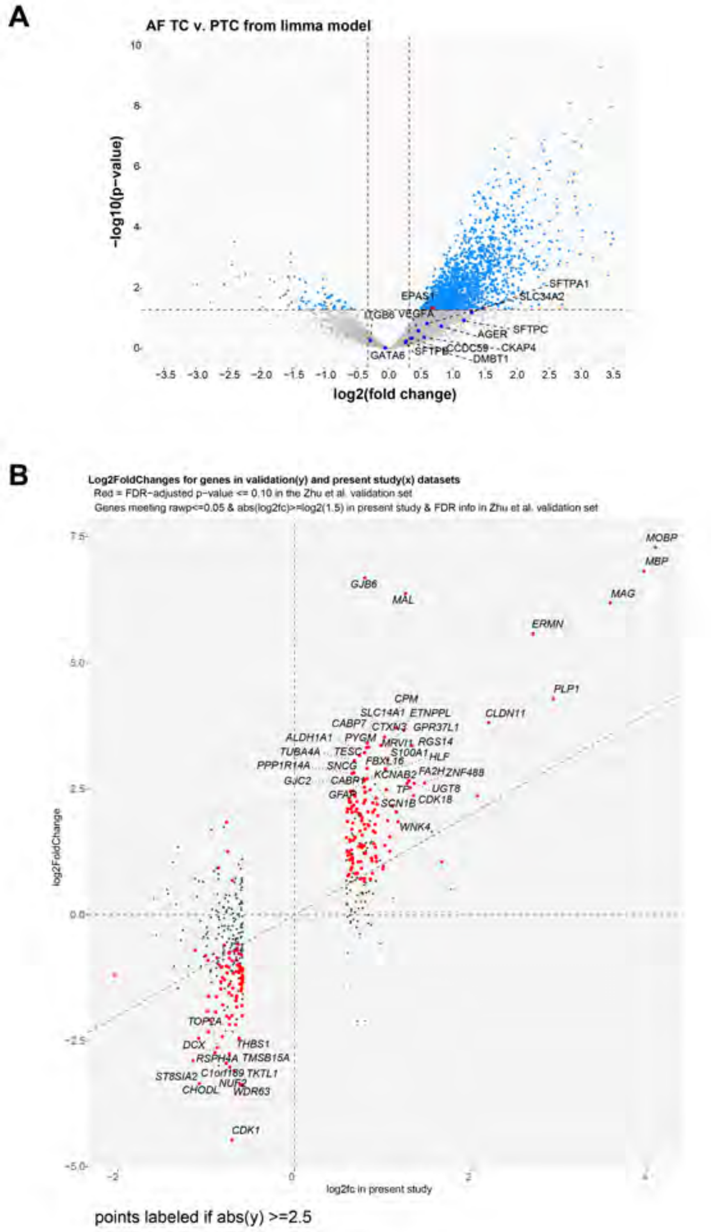

Supplementary Figure 4. Developmental programs detected in amniotic fluid by differential expression analysis. A) Volcano plot of differentially genes comparing the limma p-value and fold-change for all genes in term amniotic fluid term versus preterm controls. Prior defined human term induced genes from amniotic fluid, are highlighted red (significant) or blue (non-significant) (fold ≥ 1.25 and limma p-value ≤ 0.05 adjusted for treatment year effects). B) Scatter plot of log2 fold changes for genes considered differentially expressed in the profiled hippocampus term control sample versus preterm controls (X-axis) versus those obtained from an independent study (Zhu et al. 2018). Genes highlighted in red were considered consistently differential in both studies. n = 35 animals for amniotic fluid and 46 animals for hippocampus.

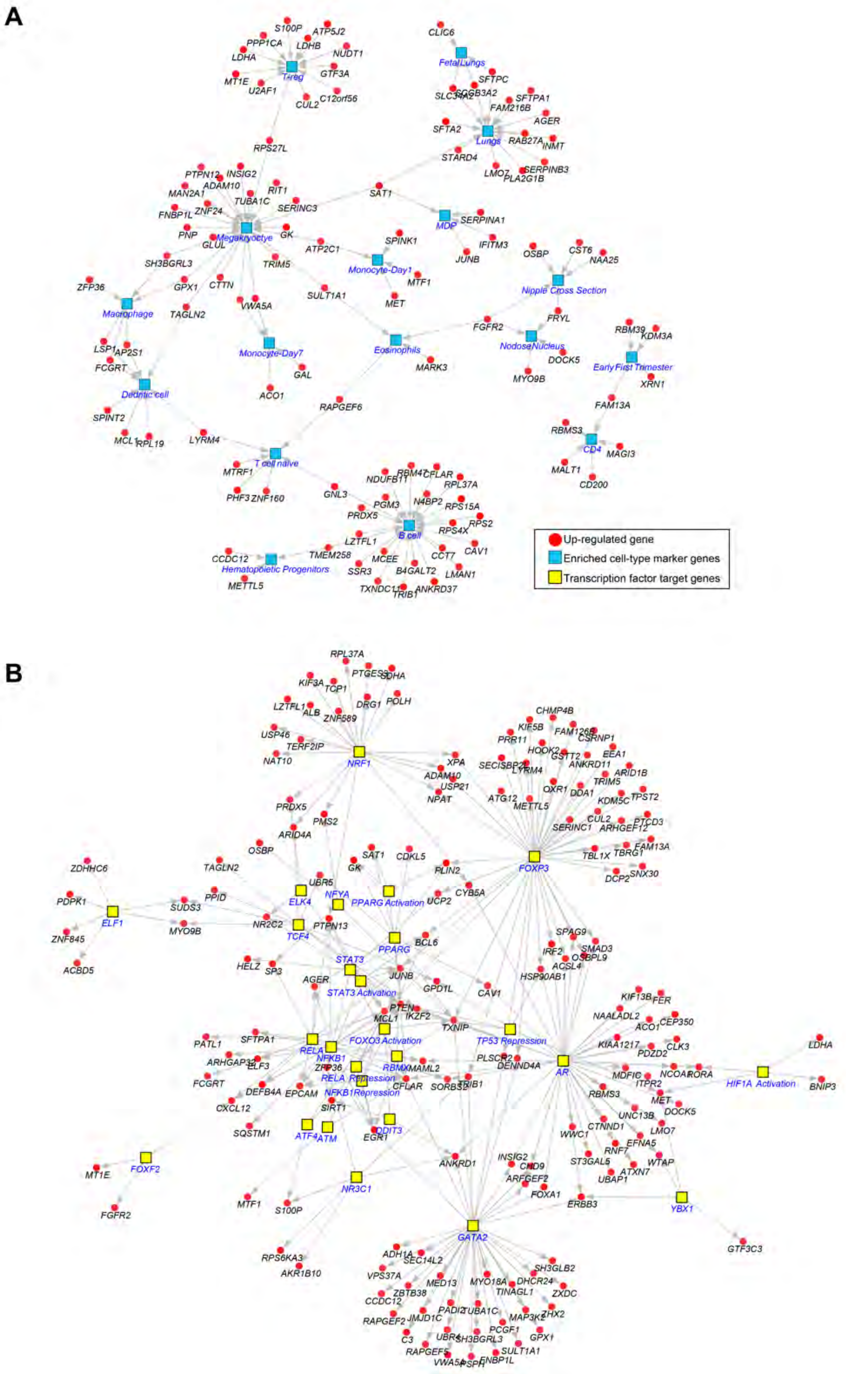

Supplementary Figure 5. Shared gestationally induced genes from lung and amniotic fluid impinge upon immune activation and associated transcription factors. A-B) GO-Elite enrichment networks, indicating each enriched gene-set (central square node) for positively correlated transcripts in the same animals (red) in lung and amniotic fluid. Blue nodes denote tissue or cellular markers (AltAnalyze TissueMarker database) (A), and yellow nodes denote putative regulatory transcription factors (B). For predicted transcription factor targets, interactions from the PAZAR and Amadeus databases was used (GO-Elite). For tissue-specific genes, the AltAnalyze TissueMarker database was used, with markers defined using the MarkerFinder algorithm applied to diverse tissues and purified cell-types (Kamath-Rayne et al., 2015). n = 35 animals.

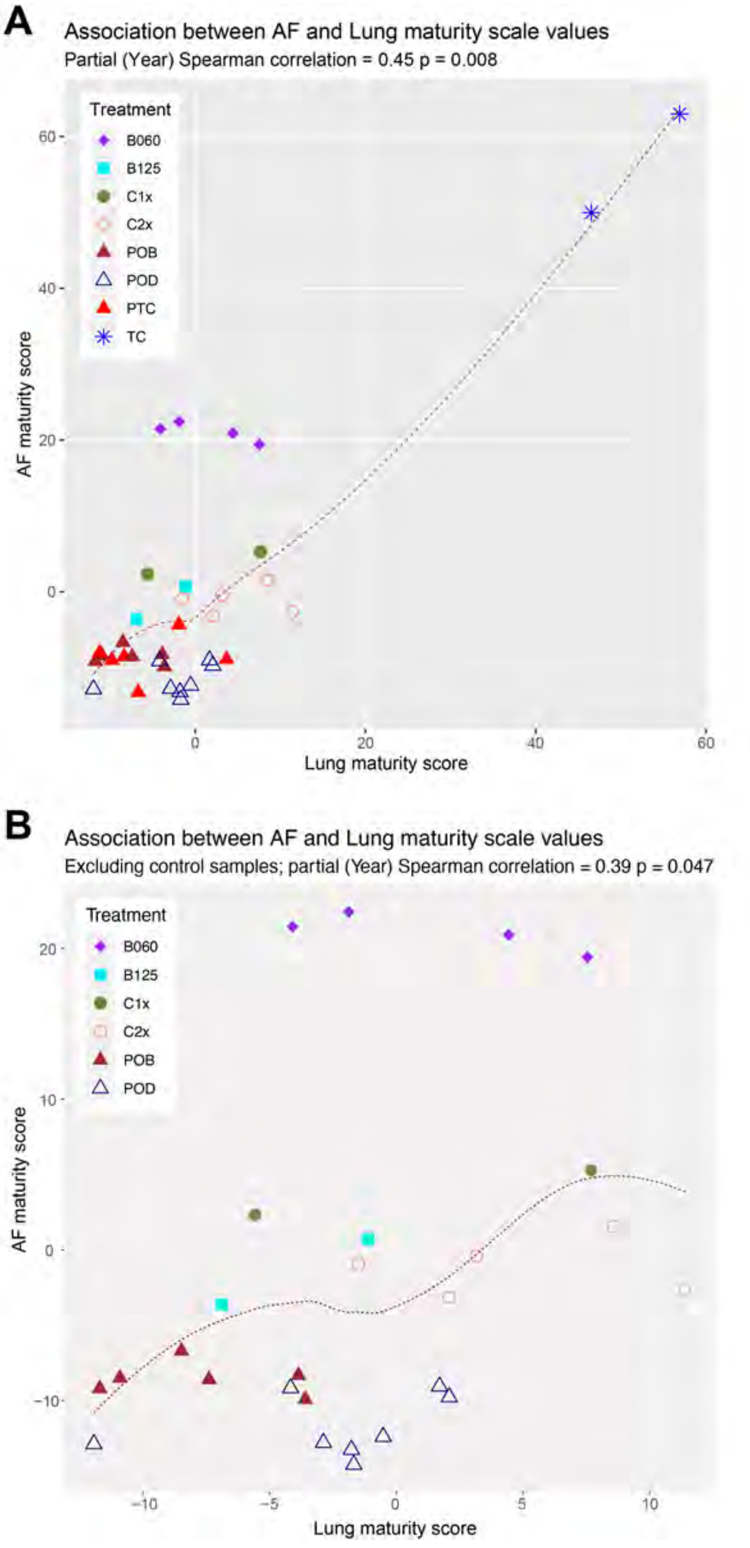

Supplementary Figure 6. Correlation of RNA Maturity Metric scores in lung and amniotic fluid. A-B) Scatter plot of computed lung maturity scores using Rhesus lung RNA-Seq with the lung model (x-axis) and amniotic fluid maturity scores from Rhesus amniotic fluid RNA-Seq with the amniotic fluid model (y-axis). A) Inclusion of all samples. B) Exclusion of term and preterm controls. n = 47 animals for lung and 35 animals for amniotic fluid.

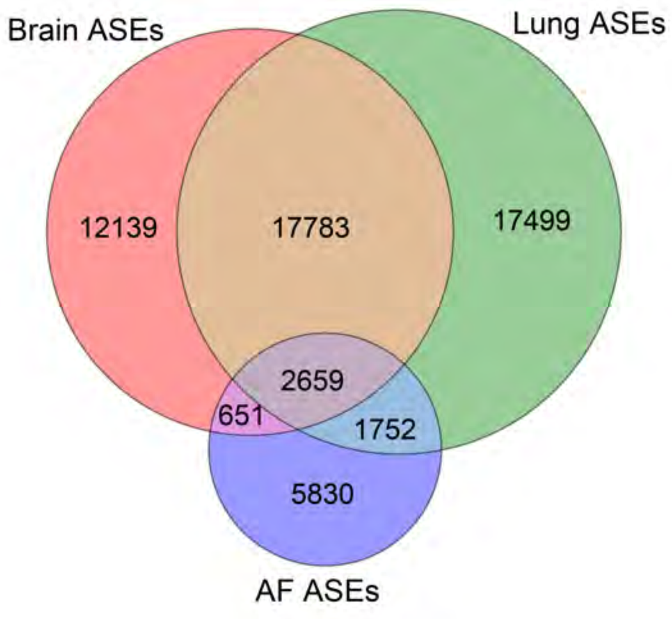

Supplementary Figure 7. Shared and unique detected splicing events in tissues and amniotic fluid. Venn diagram reporting the number of splicing-events reported from the software AltAnalyze (MultiPath-PSI), shared among the two profiled tissues (hippocampus and lung) and amniotic fluid, detected in greater than 50% of all tissue samples. Reported splicing events include known and novel events (e.g., intron retention). n = 47 animals for lung, 35 animals for amniotic fluid and 46 animals for hippocampus.
